# PCDH17 induces colorectal cancer metastasis by destroying the vascular endothelial barrier

**DOI:** 10.1038/s41419-025-07355-z

**Published:** 2025-01-21

**Authors:** Fengyun Dong, Pinghui Zhou, Feifei Kong, Sijie Cao, Xiaozao Pan, Shujing Cai, Xinke Chen, Sen Wang, Na Li, Baoyu He, Rou Zhao, Bin Zhang, Qingli Bie

**Affiliations:** 1 Department of Laboratory Medicine, Affiliated Hospital of Jining Medical University, Jining Medical University, Jining, Shandong China; 2 Postdoctoral Mobile Station of Shandong University, Jinan, Shandong China; 3 Department of Clinical Medicine, Jining Medical University, Jining, Shandong China; 4 Department of Pediatrics, Affiliated Hospital of Jining Medical University, Jining Medical University, Jining, Shandong China; 5 Department of Medical Research Center, Affiliated Hospital of Jining Medical University, Jining Medical University, Jining, Shandong China; 6 Institute of Forensic Medicine and Laboratory Medicine, Jining Medical University, Jining, Shandong China

**Keywords:** Metastasis, Prognostic markers

## Abstract

Compromised vascular integrity facilitates the cancer cells extravasation and metastasis. However, the mechanisms leading to a disruption in vascular integrity in colorectal cancer (CRC) remain unclear. In this study, PCDH17 expression was higher in the vascular endothelial cells of colon cancer with distant metastasis, and the rates of PCDH17^+^ endothelial cells (ECs) was associated with the M stage in clinical pathological characteristics analysis and correlated with a poor survival prognosis. The liver and lung metastatic dissemination of MC-38 was significantly decreased in PCDH17^–/–^mice. The ubiquitination and degradation of VEGFR2 was prevented by the interaction between PCDH17 and the E3 ubiquitin ligase MARCH5, which causing the separation of internalized VE-cadherin, and increased the vascular permeability and metastasis of CRC. These results highlight the importance of PCDH17 in maintaining vascular integrity, which has emphasis for endothelial barrier function in metastatic cancer. PCDH17 has the potential to be a marker for predicting tumor metastasis as well as a viable treatment target for CRC.

## Introduction

Colorectal cancer (CRC) is the third most common type of cancer worldwide, liver metastasis of colorectal cancer (CRCLM) is the leading cause of death. About 20% of CRC patients develop distant metastases, most commonly in the liver and lungs [1]. Intestinal venous blood travels directly to the liver via the portal vein, which is the most common hematogenous route for the transmission of CRC cells [2]. Tumor cells invade the perivascular extracellular matrix, disrupt the endothelial barrier and enter the bloodstream to metastasise. The integrity of the endothelial barrier is a major factor limiting the invasion of tumor cells through the circulatory system [3]. Therefore, exploring the molecular mechanisms involved in maintaining vascular barrier integrity could improve therapeutic interventions to prevent the distant metastasis of tumor cells.

Damage to the intestinal vascular barrier has been found to facilitate the spread of colorectal cancer to the liver. The plasma membrane vesicle-associated protein-1 (PV-1), a vascular endothelium-specific transmembrane protein, could be used to assess the damage of the intestinal vascular barrier and the increase in vascular permeability [2]. Studies have shown that HeLa cell-derived exosomes can induce endothelial cell endoplasmic reticulum stress, leading to a reduction in the expression of tight-junction-related proteins and disruption of vascular integrity, thereby promoting tumor metastasis [4]. In addition, vascular endothelial growth factor secreted by tumor cells triggers the degradation of Nrdp1 in vascular endothelial cells, which results in increased secretion of kinase Fam20C, destroys the integrity of the vascular basement membrane, and ultimately promote the metastasis of cancer cells [5]. These findings highlight that certain proteins in tumor endothelial cells are crucial for maintaining the integrity of the endothelial cell barrier. Identifying specific proteins in tumor endothelial cells that regulate barrier integrity and elucidating their regulatory mechanisms could potentially unveil novel targets for combating tumor metastasis.

Endothelial junction complexes, including adherens junctions and tight junctions, are essential in maintaining the physical barrier of blood vessels [6]. Protocadherin 17 (PCDH17) belongs to the cadherin superfamily [7, 8], and its gene is located on chromosome 13q21.1 in humans [9]. Evidence indicates that PCDH17 is crucial in numerous biological processes, including the cell cycle, apoptosis, autophagy, and signal transduction [10–12]. However, there is no mechanistic data on the role of PCDH17 in modulating the tumor vascular endothelial barrier and promoting distant metastasis of CRC. In the present study, using single-cell sequencing, we showed that PCDH17 was highly and selectively expressed in vascular endothelial cells of colon cancer tissues compared to normal vascular endothelium, and demonstrated that PCDH17 could regulate the metastatic spreading of tumor cells by disrupting the integrity of the endothelial barrier.

To date, there are few clinical markers for predicting the integrity of the vascular barrier in CRC patient, which could be used to determine the potential for tumor metastasis. In this study, we demonstrated that PCDH17 expression was elevated in the vascular endothelial cells of colon cancer with distant metastasis. The results using a splenic injection liver metastasis model showed that PCDH17 knockdown significantly decreased the metastasis of tumors to the liver and lungs, indicating that PCDH17 in endothelial cells is closely related to distant metastasis in colon cancer. Our investigation focused on how PCDH17 influences endothelial junctions and contributes to the breakdown of vascular barriers. We demonstrated that PCDH17 interacts with MARCH5, an E3 ubiquitin ligase, to block the ubiquitination and degradation of VEGFR2, resulting in the internalization of VE-cadherin and its separation from catenins. Collectively, our data indicated that PCDH17 overexpression inhibites VEGFR2 degradation, reduces vascular integrity, and enhances metastatic dissemination. Therefore, PCDH17 may serve as a valuable prognostic marker and a potential target for distal metastases.

## Results

### PCDH17 highly expressed in CRC vascular endothelial cells

**Fig. 1:**
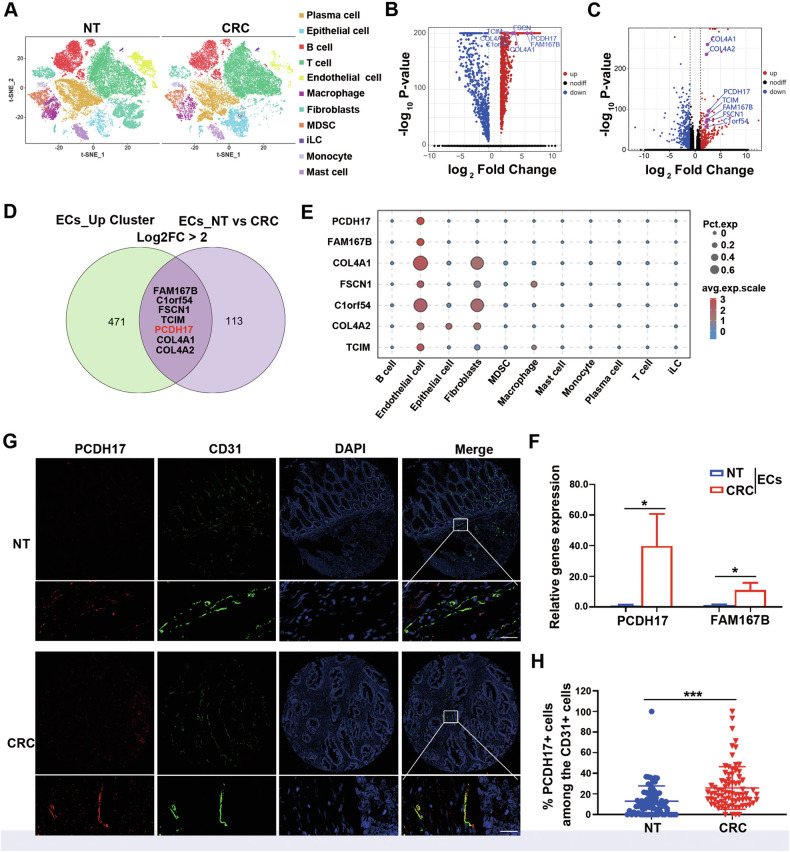
PCDH17 highly expressed in CRC vascular endothelial cells. **A** A t-distributed stochastic neighbor embedding (t-SNE) plot from adjacent or CRC tissues, grouped into 11 major cell types (*n* = 5). **B** Volcano map of DEGs in endothelial cell populations and other cell populations. **C** Volcano map of DEGs in CRC and paracancerous tissues of endothelial cell populations. The X axis represents the difference multiple value after log2 conversion, and the Y axis represents the significance value after –log10 conversion. Red and blue represents up- and downregulated DEGs, respectively, and black represents non-DEGs. (*n* = 5). **D** Overlapping candidate genes were obtained from the two differential gene signatures. **E** Bubble diagram of candidate genes expression in various cell populations. **F** RT-qPCR analysis of PCDH17 and FAM167B mRNA levels in human colon endothelial cells purified from paracancerous and CRC tissue (*n* = 3). **G** Representative immunofluorescence staining images of PCDH17 (red), CD31 (green), and DAPI (blue) in a microarray of CRCs and matched paracancerous tissues. The sections were stained by CD31 antibody to identify blood vessel lumen. Scale bars, 50 μm. **H** Percentage of cells positive for both PCDH17 and CD31 among CD31^+^ cells in CRCs and matched paracancerous tissues (n = 78). * *P* < 0.05, *** *P* < 0.001.

We conducted single-cell RNA sequencing (scRNA-seq) of tumors and the corresponding matched adjacent tissues from five patients with CRC. After quality control filtering and dimensionality reduction, we identified 11 distinct cell populations based on their specific marker genes, including one for endothelial cells (Fig. 1A). 478 genes were highly expressed in endothelial cells cluster compared to other cell clusters (> 2-fold change, *P* < 0.05; Fig. 1B) in colon tissues. In the endothelial cell populations, there were 120 up-regulated and 124 down-regulated genes between normal tissue and CRC samples (> 2-fold change, *P* < 0.05; Fig. 1C). And 7 candidate genes were identified by overlapping the two gene sets, namely *PCDH17*, *FAM167B*, *COL4A*1, *FSCN1*, *C1orf54*, *COL4A2*, and *TCIM* (Fig. 1D). Among them, *PCDH17* and *FAM167B* were specifically expressed in the tumor endothelial cells than other genes (Fig. 1E).

We further separate CD31^+^ cells from CRC tissues and corresponding adjacent normal tissues using magnetic beads to validate PCDH17 and FAM167B genes expression. And, reverse transcription-quantitative polymerase chain reaction (RT-qPCR) analyse indicated that compared with FAM167B, PCDH17 expression was significantly upregulated in vascular endothelial cells isolated from colon cancer than normal tissue (*P* < 0.05; Fig. 1F). Moreover, single-cell RNA sequencing data analysis further revealed that *PCDH17* was more specifically higher expressed in tumor endothelium than *FAM167B* (Fig. S1A). The Tumor Immunology Single Cell Centre (TISCH), a scRNA-seq database dedicated to the tumor microenvironment (TME), provides detailed single-cell level annotation. And the data generated by TISCH show that PCDH17 is almost exclusively expressed in endothelial cells (Fig. S1B), which is consistent with our results.

Immunofluorescence analysis in a CRC tissue microarrays demonstrated colocalization of PCDH17 and CD31 within vascular endothelial cells. Notably, the expression level of PCDH17 in the vascular endothelial cells of colon cancer was significantly elevated compared to that in adjacent normal colon tissues (*P* < 0.001; Fig. 1G, H). Analysis of data from The Cancer Genome Atlas (TCGA) revealed that PCDH17 expression was positively correlated with the vascular endothelial cell marker genes CD31, CD34, and von Willibrand factor (Fig. S1C). These findings suggest that PCDH17 is specifically highly expressed in neoplastic ECs and may serve as a novel biomarker of tumor endothelial cells in colon cancer.

### Endothelial PCDH17 promotes metastasis in colorectal cancer

**Fig. 2:**
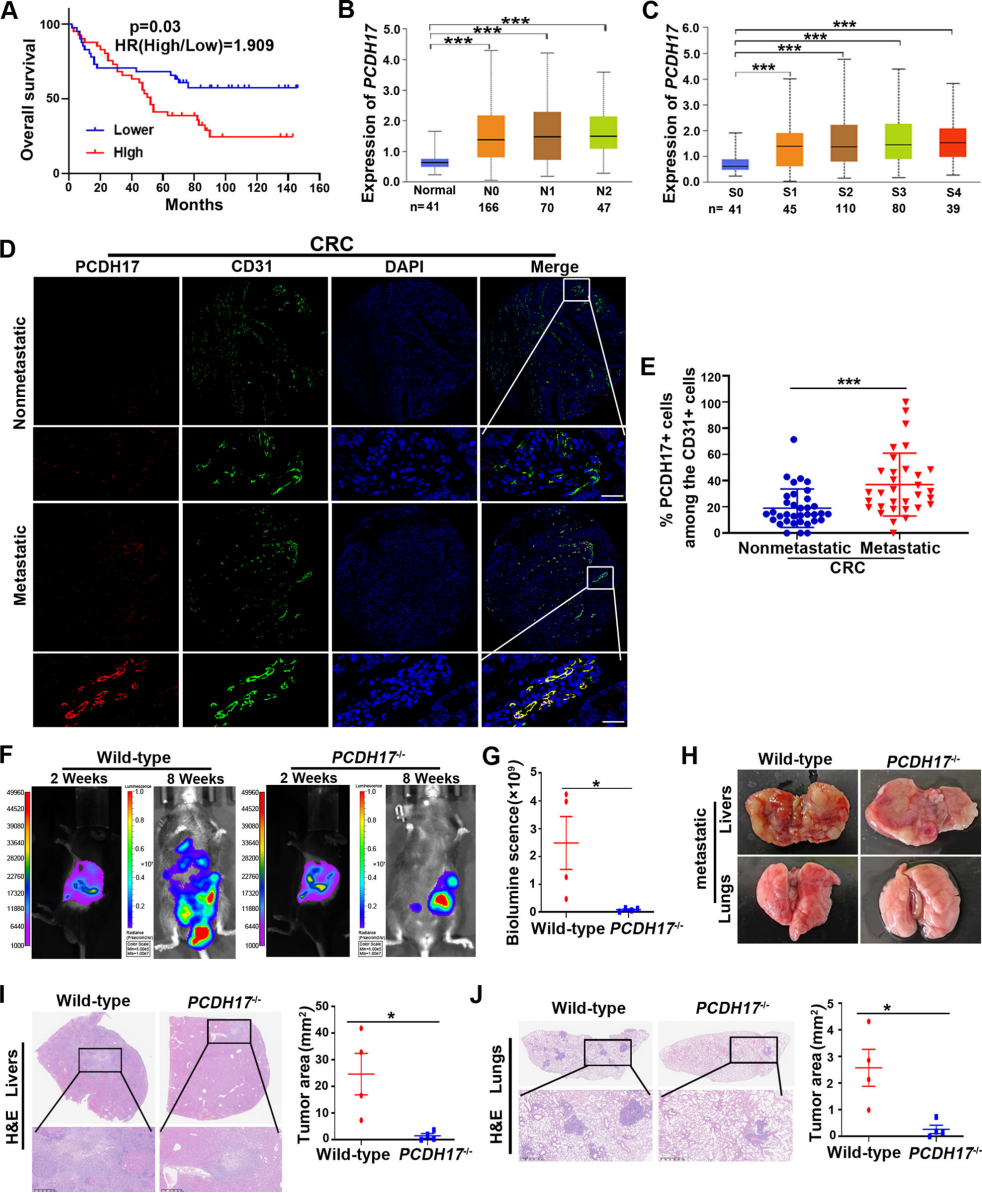
Endothelial PCDH17 promotes metastasis in colorectal cancer. **A** Survival was analyzed and compared between patients with high and low ECs expression of PCDH17 in Cohort 2 (n = 82, log-rank test, two-sided). HR, hazard ratio. **B,** **C** PCDH17 mRNA expression level by UALCAN showing (**B**) nodal metastasis and (**C**) cancer status. Differences in significance were marked (Mann-Whitney U test). Data are presented as mean ± SD. **D** Representative immunofluorescence staining images of PCDH17 (red), CD31 (green), and DAPI (blue) in a CRC tissue microarray. The microarray were stained by CD31 antibody to identify blood vessel lumen. Scale bars, 50 μm. **E** Percentage of cells positive for both PCDH17 and CD31 among CD31^+^ cells in patients with CRC with (n = 32) or without distant metastases (n = 34). **F** Bioluminescence at 14 and 56 d post-injection of MC38-luciferase. **G** Quantification of the photon flux ratio per mouse at each time point (n = 4). **H** Imaging of metastatic nodules in liver and lung specimens from metastatic models established by spleen injection of MC38 cells. **I,J** Hematoxylin and eosin staining of mouse liver or lung with metastases (Left). Quantification of the metastatic nodules area per mouse (Right) (n = 4 mice per group). * *P* < 0.05, *** *P* < 0.001.

While previous studies have indicated that PCDH17 is commonly methylated and acts as a suppressor of tumors in CRC [9], the precise role and mechanism of PCDH17 in tumor vascular endothelial cells has not been elucidated. We evaluated the correlation between PCDH17 expression in tumor vascular endothelial cells and the clinicopathological characteristics, survival prognosis using a colon cancer tissue microarray by immunofluorescence staining. Firstly, the number of CD31-positive cells and the number of PCDH17 and CD31 co-positive cells in colorectal cancer tissues were counted respectively. Then, analyze the PCDH17-positive ECs rates in ECs and the median value of PCDH17-positive ECs rates. Colon cancer tissues with PCDH17-positive ECs rates higher than the median value were defined as the high group, relative to a low group. Multivariate regression analysis demonstrated that PCDH17 expression in ECs was positively correlated with M stage (*P* = 0.002) in CRCs (Table 1). The clinical specimens statistic analysis revealed PCDH17 expression in ECs might be contribute to the metastasis of colon cancer. Moreover, high expression of PCDH17 in ECs was signifcantly associated with poor survival of CRC patients (HR = 1.909, *P* = 0.03; Fig. 2A). Further analysis of the data from TCGA revealed that PCDH17 mRNA expression in CRC tissues was also positively correlated with the N (*P* < 0.001) and clinical stages (*P* < 0.001; Fig. 2B, C), suggesting a potential association between PCDH17 expression in endothelial cells and unfavorable prognosis and distant metastasis in CRC. To further confirm that PCDH17 expression in endothelial cells participates in regulating metastasis of CRC, we next analyzed CRC tissue chips from patients with metachronous distant metastases (*n* = 32) or non-metastasest (n = 35). Immunofluorescence staining results showed the expression of PCDH17 and CD31 exist more colocalization in vascular endothelial cells of CRC tissues with metastasis than that with non-metastases (Fig. 2D). And, the proportion of PCDH17-positive ECs in endothelial cells was significantly higher in primary tumors from patients who developed distant metastases compared to those who did not (*P* < 0.001; Fig. 2 E). These results prompted that the highly expression of PCDH17 in tumor endothelial cells participates in tumor metastasis.

**Table 1:** Correlation between PCDH17 in ECs expression and clinicopathological characteristics.

Clinico-pathological features		Case	PCDH17 expression in ECs		*Χ*^*2*^ value	*P* value
			low	high		
Gender	Female	34	17	17	0.000	>0.999
	Male	48	24	24		
Age	≤60	46	24	22	0.198	0.656
	>60	36	17	19		
Grade	Well differentiation	5	3	2	0.215	0.898
	Moderate differentiation	69	34	35		
	poor differentiation	8	4	4		
T stage	T1-2	17	11	6	1.855	0.173
	T3-4	65	30	35		
N stage	N0	50	28	22	1.845	0.174
	N1-3	32	13	19		
M stage	M0	44	29	15	9.612	0.002
	M1	38	12	26		
Tumour Diameter(cm)	≤4	45	23	22	0.049	0.824
	>4	37	18	19

The liver and lungs are the most frequent sites for CRC metastasis. To evaluate the effects of PCDH17 expressed on ECs on the metastasis process, we established a liver metastasis model using MC38 cells in wild-type and in PCDH17 knockout (KO) mice, we established a liver metastasis model using MC38 cells. Monitoring of metastases in the liver was conducted through in vivo bioluminescence imaging 14 days post-injection of tumor cells. A significant reduction in bioluminescence intensity was observed in the livers of the PCDH17 KO mice compared to the wild-type group (Fig. 2F, G). Following euthanasia, the liver and lung were removed and photographed, revealing widespread tumor foci throughout the liver and lungs of wild-type mouse. Conversely, the livers and lungs of PCDH17 KO mouse displayed lower tumor foci (Fig. 2H). The results of tissue hematoxylin and eosin staining showed that a higher number of metastatic lesions in the wild-type group, while the PCDH17 KO mouse livers maintained normal structure with significantly fewer metastatic lesions (*P* < 0.05; Fig. 2I). Lung metastasis in these mice showed the same trend as liver metastasis (*P* < 0.05; Fig. 2J). These findings suggest a potential association between PCDH17 expression in ECs and tumor distant metastasis in CRC.

### PCDH17 does not affect the proliferation, migration, and tubule formation of endothelial cells in vitro

**Fig. 3:**
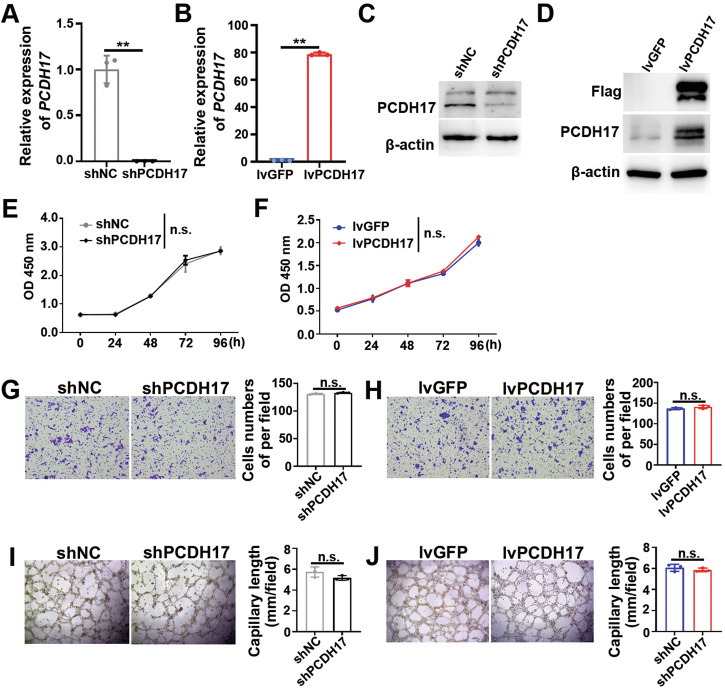
PCDH17 does not affect the proliferation, migration, and tubule formation of endothelial cells in vitro. **A-D** RT-qPCR or western blotting analysis of PCDH17 expression in the HUVECs stably transduced with PCDH17-targeting shRNA (**A, C**) and in the HUVECs stably overexpressing by lentivirus‐PCDH17 (**B, D**) (*n* = 3 technical replicates). **E,** **F** CCK-8 assays to determine the proliferation of HUVECs overexpressing or with knocked down PCDH17. **G,** **H** Transwell filter migration assays to determine the effect of PCDH17 knockdown or overexpression on the migration ability of HUVECs. **I,** **J** Tube formation assays to determine the effect of PCDH17 knockdown or overexpression on the tubule formation ability of HUVECs. n.s. no significant, ** *P* < 0.01.

To verify the role of PCDH17 in endothelial cells, loss- and gain-of-function assays were performed. Firstly, human umbilical vein endothelial cells (HUVECs) were transfected with PCDH17 shRNA lentivirus vector or PCDH17 expression lentivirus vector, or control vectors. The results of RT-qPCR and western blotting analysis indicated a significant reduction of PCDH17 levels in the shPCDH17 group compared to the control group (*P* < 0.01; Fig. 3A, C), and a significant increase of PCDH17 levels in the PCDH17 overexpression group compared to the control group (*P* < 0.01; Fig. 3B, D). Interestingly, neither PCDH17 knockdown nor overexpression had a substantial impact on endothelial cell proliferation, migration, or tubule formation, as demonstrated in Fig. 3E–J. These results revealed that PCDH17 expression in endothelial cells had no effects on the ability of proliferation, migration, and angiogenesis of endothelial cells. Next, we will explore how the expression of PCDH17 in endothelial cells influence the tumor metastasis.

### PCDH17-induced vascular endothelial barrier damage

**Fig. 4:**
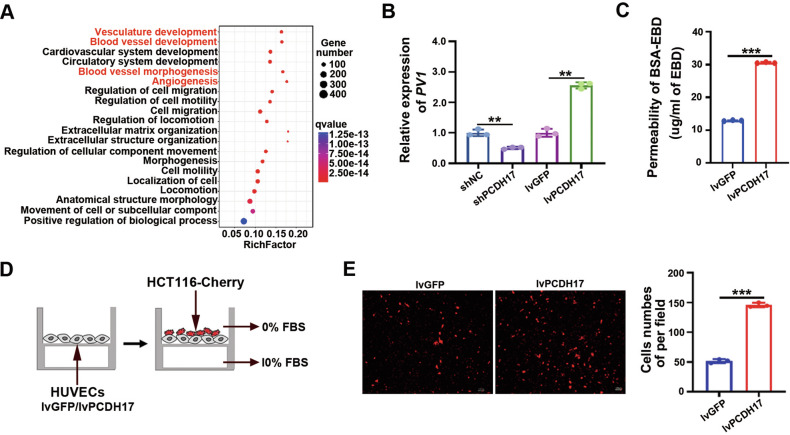
PCDH17 induced vascular endothelial barrier damage. **A** Bubble map of top 20 pathways for all DEGs by GO enrichment analysis. **B** The mRNA expression of PV1 were detected by RT-qPCR in HUVECs overexpressing or with or knocked down PCDH17. **C** Diffusion of Evans blue dye (EBD; 0.67 mg/ml) across membranes with pore diameters of 3 µm, with a monolayer of the HUVECs overexpressing PCDH17, at 3 h postdiffusion onset in the presence of BSA (40 mg/ml). The concentration of Evans blue dye that diffused across the membrane was quantified by a microplate reader, and the data are presented as the mean values (*n* = 3). **D** Transendothelial migration assay: mCherry-HCT116 cells were seeded on HUVECs confluent monolayers infected with lvPCDH17 or lvGFP. **E** Representative merges of the mCherry-positive transmigrated cells (Left). Number of mCherry-positive transmigrated cells (Right). Results are representative of three independent experiments each group in triplicate and values are expressed as the mean ± SD. **P < 0.01, ***P < 0.001.

Differentially expressed gene (DEG) enrichment was performed on PCDH17-positive and negative cell populations by single-cell sequencing analysis. The GO molecular function analysis showed that the differentially expressed genes (DEGs) mainly focused on the functions of vasculature development, blood vessel development, circulatory system development, blood vessel morphogenesis, and angiogenesis. This implies that PCDH17 is involved in vascular remodeling (Fig. 4A). Studies have confirmed that tumor vascular leakage can directly enhance tumor cell metastasis [13]. The levels of plasmalemma vesicle-associated protein PLVAP (PV-1), a marker of intestinal barrier damage [2], was significantly downregulated by PCDH17 knockdown and was upregulated by PCDH17 overexpression (Fig. 4B). Evans blue cell permeability experiments showed that PCDH17 increased endothelial cell permeability (P < 0.001; Fig. 4C), which is one of the direct causes of intestinal vascular barrier damage [14]. Additionally, a tumor transendothelial migration assay demonstrated that PCDH17 facilitates the extravasation and metastasis of HCT116 cells (*P* < 0.001; Fig. 4D, E). These findings suggest that the upregulation of PCDH17 induces an increase in endothelial cell permeability, leading to impairment of the vascular barrier function.

### PCDH17 increased vascular permeability by promoting VEGFR2 protein stability and VE-cadherin internalization

**Fig. 5:**
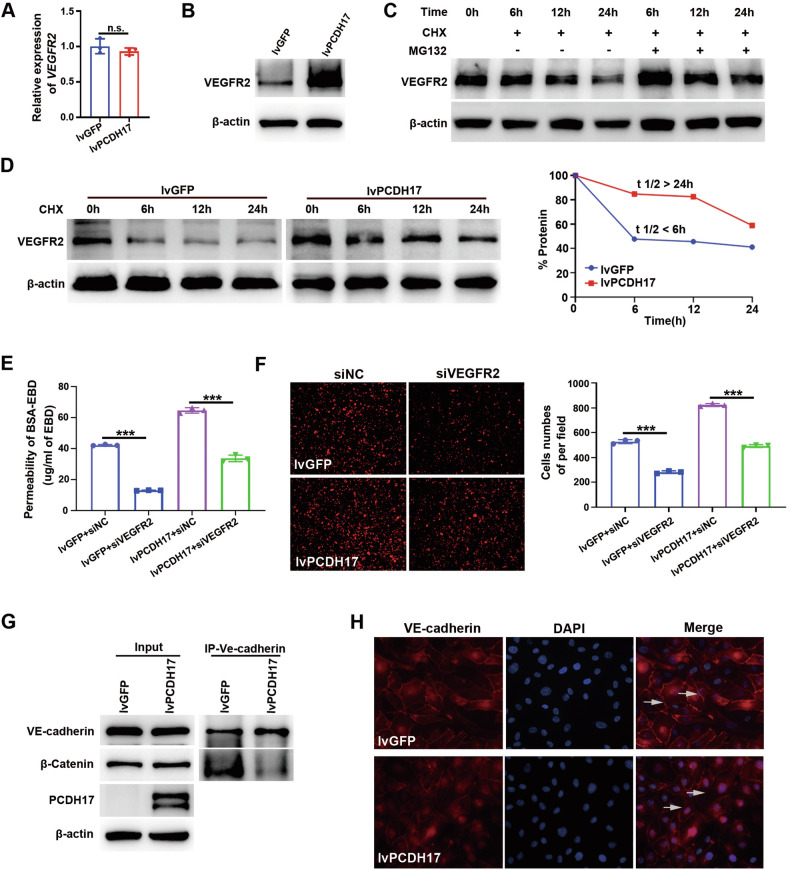
PCDH17 affects the protein stability of VEGFR2 and promotes vascular permeability through VE-cadherin. **A, B** RT-qPCR and western blotting analysis of VEGFR2 expression in HUVECs overexpressed PCDH17. **C** Representative blots showed that MG132 blocks the degradation of VEGFR2 in HUVECs overexpressing PCDH17. **D** Representative blots and quantification showing that overexpression of PCDH17 increased the stability of VEGFR2. **E** Diffusion of Evans blue dye (EBD; 0.67 mg/ml) across membranes with pore diameters of 3 µm, with a monolayer of the HUVECs overexpressing PCDH17 treated with siVEGFR2, at 3 h postdiffusion onset in the presence of BSA (40 mg/ml). The concentration of Evans blue dye that diffused across the membrane was quantified by a microplate reader, and the data are presented as the mean values (*n* = 3). **F** Representative merges of the mCherry-positive transmigrated cells (Left) crossing a monolayer of the HUVECs overexpressing PCDH17 treated with siVEGFR2. Number of mCherry-positive transmigrated cells (Right) (*n* = 3). **G** Co-IP assay showing that PCDH17 blocks the binding of VE-cadherin to β-catenin. **H** Representative merges of HUVECs stained for DAPI (blue) and VE-cadherin (red). CHX, cycloheximide. n.s. no significant, ***P < 0.001.

VEGF/VEGFR2 signaling induces vascular hyperpermeability [15]. We analyzed whether the expression of PCDH17 in ECs influence VEGF/VEGFR2 signaling. We found knockdown or overexpression the PCDH17 in HUVECs had almost no impact on mRNA expression of VEGFR2 (Fig. 5A; Fig. S2A). However, the protein level of VEGFR2 was notablely increased with PCDH17 overexpression, while a decrease was observed in the shPCDH17 HUVECs (Fig. 5B; Fig. S2B). This prompted us to speculate that PCDH17 might regulate VEGFR2 protein stability rather than affecting VEGFR2 mRNA transcription. The findings from the protein half-life experiment indicated the use of MG132 inhibited the degradation of VEGFR2 triggered by cycloheximide and overexpression of PCDH17 in HUVECs obviously inhibited the degradation of VEGFR2 (Fig. 5C, D), suggesting that PCDH17 increases the stability of VEGFR2. To confirm whether VEGFR2 is a downstream molecule in the permeability regulation by PCDH17, we knocked down VEGFR2 by siRNA in HUVECs with stable overexpression of PCDH17 (*P* < 0.05, *P* < 0.01; Fig. S2C). Evans blue cell permeability experiments showed that the increased permeability due to PCDH17 upregulation was abolished in HUVECs treated with siVEGFR2 (*P* < 0.001; Fig. 5E). Furthermore, the tumor transendothelial migration assay also demonstrated that PCDH17 facilitates the extravasation and metastasis of HCT116 cells was abolished in HUVECs treated with siVEGFR2 (*P* < 0.001; Fig. 5F). These results showed that PCDH17 increased vascular ECs permeability by promoting the protein stability of VEGFR2.

Endothelial junction complexes, including adherens junctions and tight junctions, are critical for preserving the endothelial barrier’s integrity [6, 16]. Transcriptome sequencing and immunofluorescence results showed that PCDH17 had no significant effect the expression of tight junction protein and the localization of cytoskeletal proteins (Table S1; Fig. S2D, E). Thus, we speculated that adherens junctions might be participated in the increased vascular endothelial cell permeability induced by PCDH17. VE-cadherin, a vital molecule in the adherens junctions between vascular endothelial cells, plays a crucial role in connecting to the cytoskeleton through the catenin family [17]. VEGFR2 activation by VEGFA destabilized adherens junctions, leading to VE-cadherin tyrosine phosphorylation and internalization [15]. By destroying the VE-cadherin-β-catenin complex, VEGFA weakens adhesion protein connections and induces the destruction of vascular endothelial integrity, thus promoting cancer cell extravasation and metastasis [18]. Hence, we evaluate whether the increased stability of VEGFR2 induced by PCDH17 in ECs regulate the internalization of VE-cadherin. Co-immunoprecipitation results showed that PCDH17 blocked the combination between VE-cadherin and β-catenin (Fig. 5G). And, the results from immunofluorescence staining highlighted a significant reduction in the accumulation of VE-cadherin on cell membrane of HUVECs with enhanced PCDH17 over-expression (Fig. 5H). These results indicate that PCDH17 may increase endothelial permeability by enhancing the protein stability of VEGFR2.

### PCDH17 competitively binding to the E3 ubiquitin ligase MARCH5 with VEGFR2

**Fig. 6:**
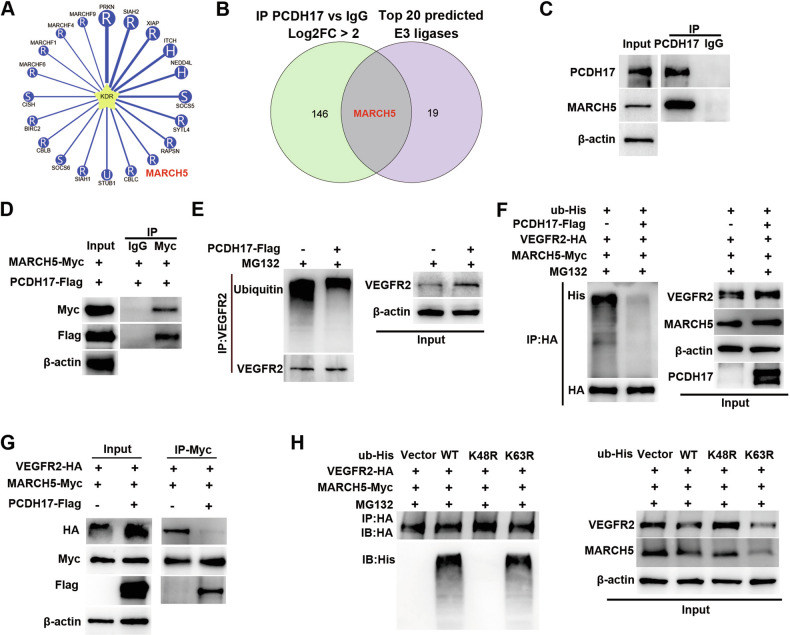
PCDH17 competitively binding to the E3 ubiquitin ligase MARCH5 with VEGFR2. **A** The ubiquitinase and substrate website predicts that MARCH5 is predicted to be a ubiquitin ligase of VEGFR2 using the UbiBrowser database. **B** Venn diagram of the overlapping predicted E3 ligase and proteins of IP-MS. **C** Co-IP assay showing that PCDH17 binding to MARCH5 in HUVECs. **D** Co-IP assay showing that 3×Flag-tagged PCDH17 interacts with Myc-tagged MARCH5 in HEK293T cells. **E** Representative blots of ubiquitination of VEGFR2 in HUVECs infected with or without 3×Flag-tagged PCDH17. **F** Representative blots of ubiquitination of VEGFR2 in HEK293T infected with or without 3×Flag-tagged PCDH17. **G** Co-IP assay to detect the interaction between PCDH17, MARCH5 and VEGFR2 in HEK293T cells. **H** MARCH5 ubiquitinates VEGFR2 through Lys48-linked ubiquitin chains in HEK293T cells and the effects of transfection with mutant HA-ubiquitin vectors (K48R and K63R).

Our previous studies found post-translational modifications of proteins were critical for their biological functions [19, 20]. Ubiquitinated degradation of VEGFR2 plays an important role in regulating endothelial function [21]. We firstly analyzed E3 ligases or deubiquitinating enzymes related to VEGFR2 in the UbiBrowser database [22], and identified the top 20 predicted E3 ligases (Fig. 6 A). To investigate how PCDH17 regulates the stability of VEGFR2, immunoprecipitation followed by mass spectrometry (IP-MS) experiments were performed to identify proteins interacting with PCDH17 and found 147 proteins bind to PCDH17 (Log2*FC* > 2) (Table S2). MARCH5 was identified by overlapping 147 proteins that bind to PCDH17 and the top 20 predicted E3 ligases related to VEGFR2 (Fig. 6 B). MARCH5 (MARCHF5, RNF153) is a mitochondrial E3 ubiquitin ligase located in the outer mitochondrial membrane that regulates endothelial cell function to protect against ischemia and hypoxia [23]. A co-immunoprecipitation assay was used to detect protein binding. Immunoprecipitation using PCDH17 antibody-coated magnetic beads showed that MARCH5 interacted with PCDH17 in HUVECs (Fig. 6C), the same results were obtained in HEK293T cells exogenously transfected with Myc-MARCH5 plasmids and PCDH17-Flag adenovirus (Fig. 6D). We speculate that PCDH17 may regulate ubiquitination degradation of VEGFR2 through MARCH5. To validate this theory, we evaluated the ubiquitination of VEGFR2 in HUVECs. The findings confirmed that the ubiquitination of VEGFR2 was suppressed when PCDH17 was overexpressed in HUVECs (Fig. 6E). Furthermore, Myc-MARCH5 and HA-VEGFR2 plasmids with or without PCDH17-Flag adenovirus co-transfected into HEK293T cells, also showed that PCDH17 blocked the ubiquitin-mediated degradation of VEGFR2 (Fig. 6F). Immunoprecipitation using Myc antibody-coated magnetic beads revealed that PCDH17 effectively blocked the binding of MARCH5 to VEGFR2 by competitively combine with MARCH5(Fig. 6G). These results suggested that PCDH17 might combine with MARCH5 to inhibit degradation of VEGFR2.

To further elucidate how MARCH5 mediates the ubiquitination of VEGFR2, HEK293T cells were co-transfected with wild-type or mutant His-Ubiquitin vectors, along with Myc-MARCH5 and HA-VEGFR2 vectors. The levels of VEGFR2 ubiquitination were assessed through western blotting using the specific antibodies. The results showed that the K48R mutant significantly decreased the ubiquitination level of VEGFR2, indicating that polyubiquitination of VEGFR2 predominantly involves Lys48-linked chains (Fig. 6H).

## Materials And Methods

### Antibodies and plasmids

PCDH17 (PA5-55428) antibody for immunofluorescence was bought from Thermo Fisher Scientific (Waltham, MA, USA). β-actin (81115-1-RR), Myc (16286-1-AP), Flag-tag (80010-1-RR), HA-tag (51064-2-AP), His-tag (66005-1-Ig), and β-catenin (6379-1-Ig) antibodies were purchased from Proteintech (Rosemont, IL, USA). VE-cadherin (AF6265) and VEGFR2 (AF6281) antibodies were purchased from Affinity Biosciences (Cincinnati, OH, USA). PCDH17 (ab128815) and CD31 (ab9498) antibodies were purchased from Abcam (Cambridge, UK). Myc-vector, HA-vector, Myc-MARCH5, and HA-VEGFR2 plasmids were purchased from Shandong Gene&Bio Co. (Shandong, China), and HA-ubiquitin (wild-type and K48R and K63R mutants) plasmids were purchased from Hunan Fenghui Biotechnology Co. (Hunan, China).

### Tissue samples

CRC tissues along with matched normal tissues were obtained by the Department of Gastroenterology at the Affiliated Hospital of Jining Medical University (Jining, China) in accordance with the codes established by the Ethical Committee of the Affiliated Hospital of Jining Medical University(Approval No. 2021B006). Before inclusion in the study, the participants provided written informed consent. Tissue microarrays (TMAs) were purchased from Superbiotek (COC1601; Shanghai, China) and included 83 CRC and adjacent normal tissues (Among them, 1 case of CRC and 5 cases of adjacent normal tissues were not included in the analysis and research scope due to negative CD31 staining). The clinical characteristics and pathological information of the participants with the TMAs are listed in Table S3.

### scRNA‑seq analysis

Gene Denovo Biotechnology Co., Ltd. (Guangzhou, China) conducted RNA library sequencing on an Illumina HiseqTM 2500/4000 for 5 pairs of CRC tissues. Visualization techniques such as t-distributed Stochastic Neighbor Embedding (tSNE) projection and Uniform Manifold Approximation and Projection (UMAP) were utilized. Cell type annotations were assigned using SingleR on Blueprint and Encode reference datasets, alongside marker-based adjustments. The cells were categorized into 11 main cell types.

### Cell culture

HUVECs, HCT116 cells, and HEK293T cells were cultured in DMEM (Gibco, C11995500BT). MC38 cells were cultured in Roswell Park Memorial Institute 1640 medium (Gibco, C11875500BT). All media contained 10% (fetal bovine serum FBS (Gibco; 10099-141 C). All cells were cultured in an incubator at 37 °C with 5% CO_2_. Regular testing for mycoplasma contamination was also performed. Cell lines were authenticated by Genetica DNA Laboratories using STR proling.

### Establishment of stable infected cell lines

Recombinant lentiviruses expressing green fluorescent protein (lvGFP) or PCDH17 (lvPCDH17) and knockdown lentivirus PCDH17 (shPCDH17) or control (shNC) were obtained from Syngenbio (Beijing, China). HUVECs at 30%–50% confluence were infected with lentivirus at a multiplicity of infection of 100. We screened shPCDH17 or shNC stable knockdown and overexpression lvPCDH17 or lvGFP HUVECs cells using puromycin. PCDH17 expression was validated by RT-qPCR and western blotting analyses.

### Plasmid DNA transfection and RNA interference

Cells at 70% confluence were transfected with the indicated plasmid DNA using Lipofectamine 3000 transfection reagent (Invitrogen, L3000015) according to the manufacturer’s protocol. The siRNAs targeting the indicated genes and scramble siRNA controls were synthesized by Shandong Gene&Bio Co. Ltd (Shandong, China). Cells at 30-40% confluence were transfected with Lipofectamine 3000 (Invitrogen, L3000015). Cells were trypsinised at 48-72 h post-transfection for various assays. The sequences of siRNAs are listed in Table S4.

### Isolation of human colon cancer endothelial cells

Fresh CRC tissue with matching adjacent normal tissue was harvested and incubated in the collagenase type I (SCR103, Merck 7 Co., Inc., Rahway, NJ, USA) at 37 °C for 50 min with intermittent shaking. Cells were completely dissociated through repeated pipetting and gentle mincing. Following filtration through a 70-µm strainer, the cells were centrifuged at 1500 rpm for 5 min and washed with PBS thrice. For the purification of colon cancer endothelial cells, the filtered colon cell digest was treated with magnetic bead sorting using PECAM1 primary antibody (130-091-935, Miltenyi Biotec, Bergisch Gladbach, Germany) for 15 min at 4 °C, followed by PBS washing and placement on a magnetic rack. Endothelial cells were isolated using a magnet, and subsequent PBS washes ensured high purity (> 90%) endothelial cells. The cells were then lysed and subjected to RT-qPCR analysis.

### Western blotting

The cells were lysed utilizing a radioimmunoprecipitation assay buffer and then subjected centrifuged at 12,000 g and 4 °C for 10 min to obtain the supernatant. Equal quantities of protein were separated by SDS-PAGE and subsequently transferred onto a polyvinylidene fluoride (PVDF) membrane. Following a blocking step with 5% non-fat milk, the membranes were incubated with primary antibodies overnight at 4 °C, followed by incubation with secondary antibodies for 1.5 h at room temperature. The blots were visualized using enhanced chemiluminescence detection reagents (Millipore, Burlington, MA, USA). β-actin served as an internal control.

### RNA extraction and quantitative PCR

The cells were collected for RNA extraction. Total RNA was performed as per the instructions provided by the manufacturer using TRIzol reagent (Omega Bio-tek, Inc., Doraville, GA, USA). The obtained RNA was subsequently reverse-transcribed into complementary DNA (cDNA) utilizing the RevertAid First Strand cDNA Synthesis Kit (Fermentas K1622; Burlington, ON, Canada). RT-qPCR was conducted using an Applied Biosystems® 7500 Fast instrument (Applied Biosystems, Waltham, MA, USA), with reactions set in an Eco 96-well plate and employing hamQ Universal SYBR qPCR Master Mix (Q711, Vazyme, Nanjing, China). Each individual PCR reaction, with a final volume of 20 μl, comprised 10 μl of 2×Master mix, 0.4 μl of Primer1 (10 μM), 0.4 μl of Primer2 (10 μM), 2 μl of template cDNA and the rest made up by ddH2O. The primer sequences are listed in Supplementary Table S5.

### Transwell and tumor trans-endothelial migration assay

To investigate the effect of PCDH17 on cell migration, we established a stably transfected cell lines. Initially, 2 × 10^4^ cells were seeded in the upper chamber of a transwell system featuring 8 µm pore-sized membranes (3422; Corning, Corning, NY, USA), which were suspended in 100 μl of serum-free medium. The bottom chamber was supplied with 600 μl medium that included 20% serum. For the transmigration assay, once HUVECs formed an endothelial monolayer, 1 × 10^5^ pre-Cherry labelled HCT116 cells were seeded on top of this layer. After an incubation period of 24 h, cells were fixed within the chamber using 95% absolute ethanol for 15 min, followed by staining with 0.1% crystal violet for 30 min, and then photographed. Specifically, for the transmigration assay, HCT116-Cherry signal was observed under a fluorescence microscope and photographed.

### CCK-8 experiments

A CCK-8 assay kit (CK-04-500T, Dojindo Laboratories, Kumamoto, Japan) was used to determine the impact of PCDH17 on HUVECs proliferation. Stably infected cell (2 × 10^3^ cells in 100 μl of culture medium) were plated in 96-well plates and cultured, then incubated with CCK-8 solutions for 1 h at specified time intervals (0, 1, 2, 3, and 4 days). Following this, absorbance was read at 450 nm using a microplate reader.

### Tube formation assay

Infected HUVECs (3 × 10^4^ cells/well) were pre-cultured in serum-free medium for a duration of 6 h and seeded onto a Matrigel‐coated 96‐well plate. After 12 h, the cells were photographed.

### Cell permeability assay

Transwell permeability assays were conducted using Transwell chambers with 3 µm pore diameters (3415; Corning). Infected HUVECs were plated on transwell inserts and grown into monolayers. The culture chambers were washed with PBS, and 100 μl of serum-free DMEM containing Evans blue dye (E2129, Sigma–Aldrich, St. Louis, MO, USA) was added to the top chamber. Concurrently, 600 μl 4% BSA was added to the bottom chamber. At various times post the onset of diffusion, 100 µl of the medium from below the insert was collected, and its absorbance at 620 nm was measured using a microplate reader.

### Co-immunoprecipitation experiments and mass spectrometry

For co-immunoprecipitation (co-IP) experiments, cell lysates were obtained and an equivalent amount of protein was precleared with protein A/G magnetic beads for 1 h (B23202, Selleck Chemicals, Houston, TX, USA). Supernatants were then incubated with anti-VE-cadherin, anti-HA, anti-FLAG, anti-Myc, rabbit control IgG (A7016, Beyotime, Shanghai, China), or mouse control IgG (A7028, Beyotime) at 4 °C overnight, and the immunoprecipitated proteins were collected using protein A/G magnetic beads for 3 h at 4 °C with rotation. The immune complexes were subsequently washed three times and analyzed by western blotting using the indicated antibodies or identified by liquid chromatography-tandem mass spectrometry (LC-MS/MS).

### Ubiquitination assays

Successful transfection of HEK293T cells or HUVECs was achieved with the specified plasmids and subsequent lysis in ice-cold immunoprecipitation buffer. Likewise, proteins in cell lysates were bound to beads through overnight incubation with specified antibodies. These beads were then eluted and utilized in western blot analysis with a combination of anti-HA, anti-ubiquitin, and anti-His primary antibodies, followed by the corresponding secondary antibodies.

### Public database analysis

The prognostic value of the *PCDH17* gene in CRC was obtained from UALCAN (https://ualcan.path.uab.edu/cgi-bin/ualcan-res.pl). The correlation analysis of *PCDH17* with endothelial marker genes was extracted from GEPIA (http://gepia.cancer-pku.cn/). The expression of *PCDH17* in various cell populations of CRC tissues was obtained from TISCH (http://tisch.comp-genomics.org/home/). The E3 ubiquitin ligase activity of VEGFR2 was predicted using the Ubibrowser website (http://ubibrowser.bio-it.cn/ubibrowser_v3/home/index).

### Cycloheximide chase

The stable cell lines of HUVECs were treated with cycloheximide (50 µg/ml) for varying time periods (0, 6, 12, and 24 h). At the designated times, the cells were lysed and underwent western blotting. The quantity of protein was assessed by comparing the densitometric value of interest with that of the loading control. The degradation rate of VEGFR2 was determined by setting the zero time point as 100%. Subsequently, the VEGFR2 expression levels at other time points were divided by their corresponding zero time point, resulting in two sets of ratios for statistical analysis. The slopes of these curves reflect the rate of protein degradation.

### Immunofluorescence staining

The tissue microarrays (TMAs) were stained with PCDH17 antibody or co-stained with a CD31 antibody overnight at 4 °C. For the HUVECs, the cells were treated with 4% formaldehyde for fixation and permeabilized with Triton X-100 (P9600, Solarbio, Beijing, China) for 15 min, then incubated with specific antibody overnight at 4 °C. Following this, the sections or cells were incubated with a fluorescent secondary antibody for 1.5 h at room temperature. Finally, nuclei of the sections or cells were stained with DAPI (Roche, Mannheim, Germany), and images were photographed with a fluorescence microscope or by scanning every point of a tissue microarray using a digital full-slice pathology scanner.

### Image analysis

Using the Visiopharm software, all tissue areas were selected, all cells were intelligently identified, and the fluorescence intensity of the identified area of each cell was determined. Each cell line was counted only once and not repeated. Counting the number of positive cells, the number of PCDH17 and CD31 co-localized cells accounted for the number of CD31 positive cells, which was the positive rate of PCDH17 expression in endothelial cells.

### Mice

A PCDH17 knockout (KO) founder mouse with a C57BL/6 background was produced by GemPharmatech (Nanjing, China) using CRISPR/Cas9 technology. The 5’-UTR and exon 1, together, were selected as the target site. Two guide RNAs were designed to target sequences located before the 5’-UTR and within intron 1–2. For the production of the KO mouse, Cas9 mRNA and guide RNA obtained by in vitro transcription were injected into fertilized embryos. Genotyping of the founders was carried out using PCR, followed by DNA sequencing analysis. Positive founders were then bred to produce offspring, which were also genotyped by PCR and DNA sequencing. The PCR primers can be found in Supplementary Table S6.

All procedures involving animals were conducted in line with the animal experimentation and welfare established by the Ethical Committee of the Affiliated Hospital of Jining Medical University (Approval No. 2021B006).

### Experimental metastasis

In order to develop animal models of CRC with distant metastasis, six-week-old female PCDH17 KO and wild type (WT) mice were used for in vivo research. We injected 5×10^5^ MC38-LUC cells (suspended in 100 µl of PBS) into the spleen using an insulin syringe. Fluorescence imaging was performed 2 weeks after injection using the IVIS Spectral in vivo Imaging System (IVIS Lumina System, PerkinElmer, Waltham, MA, USA). After 6 weeks, fluorescence was monitored again 8 weeks after injection using the IVIS Spectral in vivo imaging system, following which the mice were euthanized and liver and lung samples were gathered for further analysis. Sections of the liver and lungs were stained with hematoxylin and eosin to determine the number of lesions per equivalent area section. Each figure displays data from three or more distinct experiments.

### Statistical analysis

GraphPad Prism 6.0 (GraphPad Software, San Diego, CA, USA) was used for statistical analysis. Data were presented as the mean ± SD. We used a Student’s t-test (two-tailed) or chi-squared analysis to evaluate statistical differences between groups. Significance was determined at a *P*-value of less than 0.05.

## Discussion

PCDH17 is a member of the protocadherin superfamily that acts as a methylated gene and regulates apoptosis, autophagy, and drug resistance in tumor cells. However, its influence on vascular stability and metastatic dissemination has not been examined before this study. The formation of tumor metastasis is a multistep process that involves the cancer cells intravasation into the blood circulation through tumor vessels [24]. Increased vascular permeability and altered endothelial barrier properties contribute significantly to cancer metastasis [25]. The vascular barrier, composed of endothelial cells, the extracellular matrix, and pericytes, restricts metastatic dissemination [26]. In this study, PCDH17 was identified as an oncogenic factor that promotes the distant metastasis of CRC. First, PCDH17 was specifically elevated in colon cancer tumor endothelial cells and was positively correlated with clinically distant metastases. Second, we demonstrated that PCDH17 disrupts endothelial cell adhesion junctions, increases permeability, and facilitates the intravasation of CRC tumor cells. Mechanistically, we provide evidence that VEGFR2 serves as a substrate for the MARCH5 E3 ligase and that PCDH17 binds directly to MARCH5, preventing the ubiquitinated degradation of VEGFR2.

Single-cell sequencing technology is an ideal tool for uncovering the heterogeneity of tumor cells owing to its high degree of accuracy and specificity. We found that PCDH17 is the only gene specifically expressed in colon cancer endothelial cells, which is consistent with the public databases. Single-cell sequencing results proved that PCDH17 was highly expressed in the endothelial cells of the left ventricle of the mouse heart, providing support for the use of PCDH17 as a novel marker of the tumor endothelium [27]. Distant metastasis is the most common cause of mortality in patients with CRC. The results of the CD31 and PCDH17 double-stained tissue microarray showed that the expression of PCDH17 in the vascular endothelium of colon cancer with distant metastasis was higher than that in colon cancer without metastasis, and PCDH17 in colon cancer vascular endothelial cells may be involved in the distant metastasis of colon cancer. Furthermore, PCDH17 KO mice had significantly reduced liver and lung metastases of tumor cells compared to wild-type mice.

PCDH17 is involved in proliferation [12], apoptosis [9], and drug resistance in tumor cells [10]. Although some studies have indicated that PCDH17 exhibits a phenomenon of inhibiting tumor progression. Low expression of PCDH17, due to its high DNA methylation in cancer cells, including ovarian cancer, hepatocellular carcinoma, bladder cancer, contributed to malignant progression of tumour [28–30], and PCDH17 expression improves the sensitivity of CRC to 5-FU treatment by inducing apoptosis and JNK-dependent autophagic cell death [10]. However, our results revealed that up-regulated PCDH17 had no effects on the proliferation, migration, and tubule formation in endothelial cells, but it could significantly promoted endothelial cell permeability and the metastasis of CRC. The differential effects of PCDH17 on tumor cells and endothelial cells might be attributed to the specific tissue structure and function of endothelial cells. Cancer-induced angiogenesis and vascular permeability are a key processes in cancer metastasis [31]. The structure of adherent junctions in the endothelial cells layer is crucial for controlling vascular permeability, preventing blood components from extravasating, and maintaining the structure of endothelial cells [18, 32]. The interplay between VEGFR2 and VE-cadherin is vital for regulating junctions between vascular endothelial cells and maintaining vascular integrity [33]. However, this interaction is disrupted by VEGF, which induces tyrosine phosphorylation of VEGFR2. The phosphorylated site (Y951) then binds to the T-cell specific junction protein, activating Src, which then phosphorylates VE-cadherin directly, dissociating it from the catenins, leading to disruption of vascular integrity [34, 35].

PCDH17, a family of non-clustered protocadherins, may possess heterophilic adhesion properties and play a relevant role as a membrane-bound ligand or integrin receptor [7]. We provided evidence that PCDH17 can bind to MARCH5, MARCH5 is identified as an E3 ubiquitin ligase responsible for the specific ubiquitination of VEGFR2. Ubiquitinated degradation of VEGFR2 plays a key role for regulating both neovascularization and endothelial barrier integrity [21]. Our data suggested that PCDH17 positively regulates VEGFR2 stability via the proteasome pathway. This biological regulatory relationship suggests that PCDH17 competitively binds to MARCH5 affecting its interaction with VEGFR2. High VEGF levels, secreted by tumor cells, trigger VEGFR2 phosphorylation, which results in VE-cadherin dissociation from catenins and increased endothelial cell permeability. Moreover, the role of VEGFR2 as a novel ubiquitination substrate for MARCH5 has not yet been reported, and the mechanism underlying the interaction between the two proteins needs to be a detailed investigation.

Our results demonstrate reduced metastasis of tumor cells to the liver and lungs in PCDH17^–/–^ mice, potentially due to reduced VEGFR2 signaling. This highlights the significant impact of vascular permeability on metastatic spread. Overall, our data identified PCDH17 as a critical role in regulating endothelial barrier function in tumors by modulating VEGFR2 activity. Improving our understanding of the interaction between PCDH17 and VEGFR2 may clarify the role of PCDH17 in CRC and support its potential use as a therapeutic target. This research has important implications for targeting PCDH17 in cancer treatment.

## Supplementary information

Supplemental Figure Legends

Supplemental Figure S1

Supplemental Figure S2

Supplemental Table S1

Supplemental Table S2

Supplemental Table S3

Supplemental Table S4

Supplemental Table S5

Supplemental Table S6

Original data

## Data Availability

Data and materials supporting the findings of this study are available from the corresponding author upon reasonable request. The original/source data of scRNA‑seq (accession number: HRA004557) and RNA-seq (accession number: subHRA012498) are deposited in the Genome Sequence Archive (GSA, https://ngdc.cncb.ac.cn/gsa/).
