# Correction: PCDH17 induces colorectal cancer metastasis by destroying the vascular endothelial barrier

**DOI:** 10.1038/s41419-025-07677-y

**Published:** 2025-05-06

**Authors:** Fengyun Dong, Pinghui Zhou, Feifei Kong, Sijie Cao, Xiaozao Pan, Shujing Cai, Xinke Chen, Sen Wang, Na Li, Baoyu He, Rou Zhao, Bin Zhang, Qingli Bie

**Affiliations:** 1 Department of Laboratory Medicine, Affiliated Hospital of Jining Medical University, Jining Medical University, Jining, Shandong China; 2 Postdoctoral Mobile Station of Shandong University, Jinan, Shandong China; 3 Department of Clinical Medicine, Jining Medical University, Jining, Shandong China; 4 Department of Pediatrics, Affiliated Hospital of Jining Medical University, Jining Medical University, Jining, Shandong China; 5 Department of Medical Research Center, Affiliated Hospital of Jining Medical University, Jining Medical University, Jining, Shandong China; 6 Institute of Forensic Medicine and Laboratory Medicine, Jining Medical University, Jining, Shandong China

**Keywords:** Metastasis, Prognostic markers

Correction to: *Cell Death and Disease* 10.1038/s41419-025-07355-z, published online 21 January 2025

Following the publication of this paper, the authors have identified an error in Figure 3G where the representative migration image of HUVECs with PCDH17knockdown (shPCDH17) was inadvertently presented with the control group’s migration image during manuscript preparation.

We sincerely apologize for this oversight and would like to clarify that this correction does not impact any of the study’s conclusions. The corrected figure now accurately represents the shPCDH17 group, ensuring data integrity and reinforcing the reproducibility of the study’s findings.

**Corrected Figure 3** 
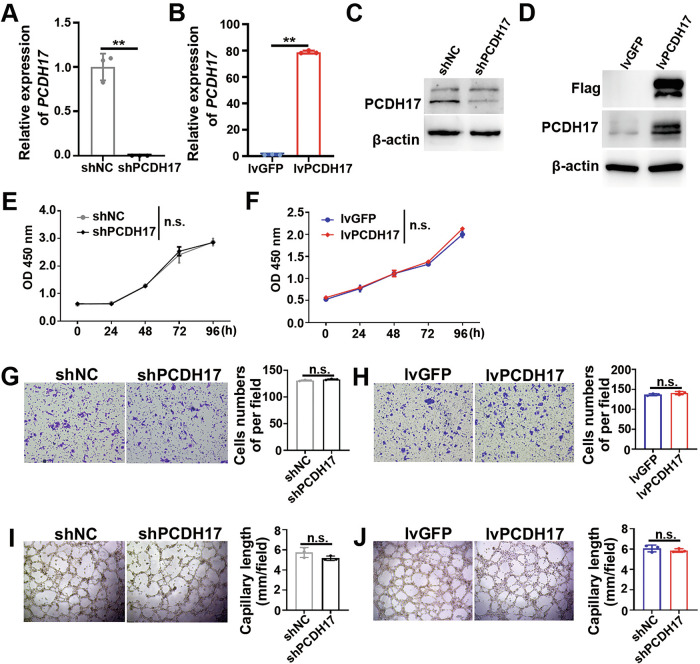


**Original published Figure 3** 
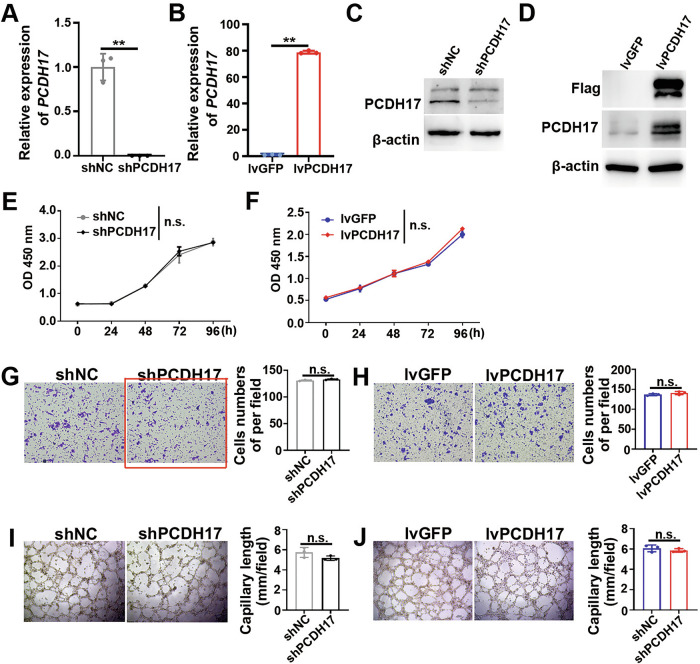


The original article has been corrected

