# Supplementary material for: PCDH17 induces colorectal cancer metastasis by destroying the vascular endothelial barrier
Source: Cell Death Dis. 2025 Jan 21;16(1):36. doi: 10.1038/s41419-025-07355-z (PMC11750977; doi:10.1038/s41419-025-07355-z)
Supplement: Supplementary file 1 — Supplemental Figure Legends [file 41419_2025_7355_MOESM1_ESM.docx]

**Supplemental Figure Legends**

**Fig. S1 PCDH17 highly expressed in CRC vascular endothelial cells. A** Violin plots of PCDH17 and FAM167 expression in paracancerous and CRC tissue. **B** TISCH website analyses of PCDH17 expression in different colon cancer databases at GSE. **C** Correlation between PCDH17 and CD31, CD34, von Willibrand factor mRNA expression in TCGA samples by GEPIA2 (Pearson correlation coefficient).

**Fig. S2** **PCDH17 does not enhance cell permeability by affecting cytoskeletal localization.** **A, B** RT-qPCR and western blotting analysis of VEGFR2 expression in HUVECs knocked down PCDH17. **C** RT-qPCR analysis of VEGFR2 expression in control (lvGFP) and PCDH17 upregulated (lvPCDH17) endothelial cells in the presence or absence of the siVEGFR2(n = 3). **D** Representative merges of HUVECs stained for DAPI (blue) and F-actin (green). **E** Representative merges of HUVECs stained for DAPI (blue) and β-tubulin (red). n.s. no significant, **P* < 0.05, ***P* < 0.01.
