# Supplementary figures and images for: PCDH17 induces colorectal cancer metastasis by destroying the vascular endothelial barrier
Source: Cell Death Dis. 2025 Jan 21;16(1):36. doi: 10.1038/s41419-025-07355-z (PMC11750977; doi:10.1038/s41419-025-07355-z)

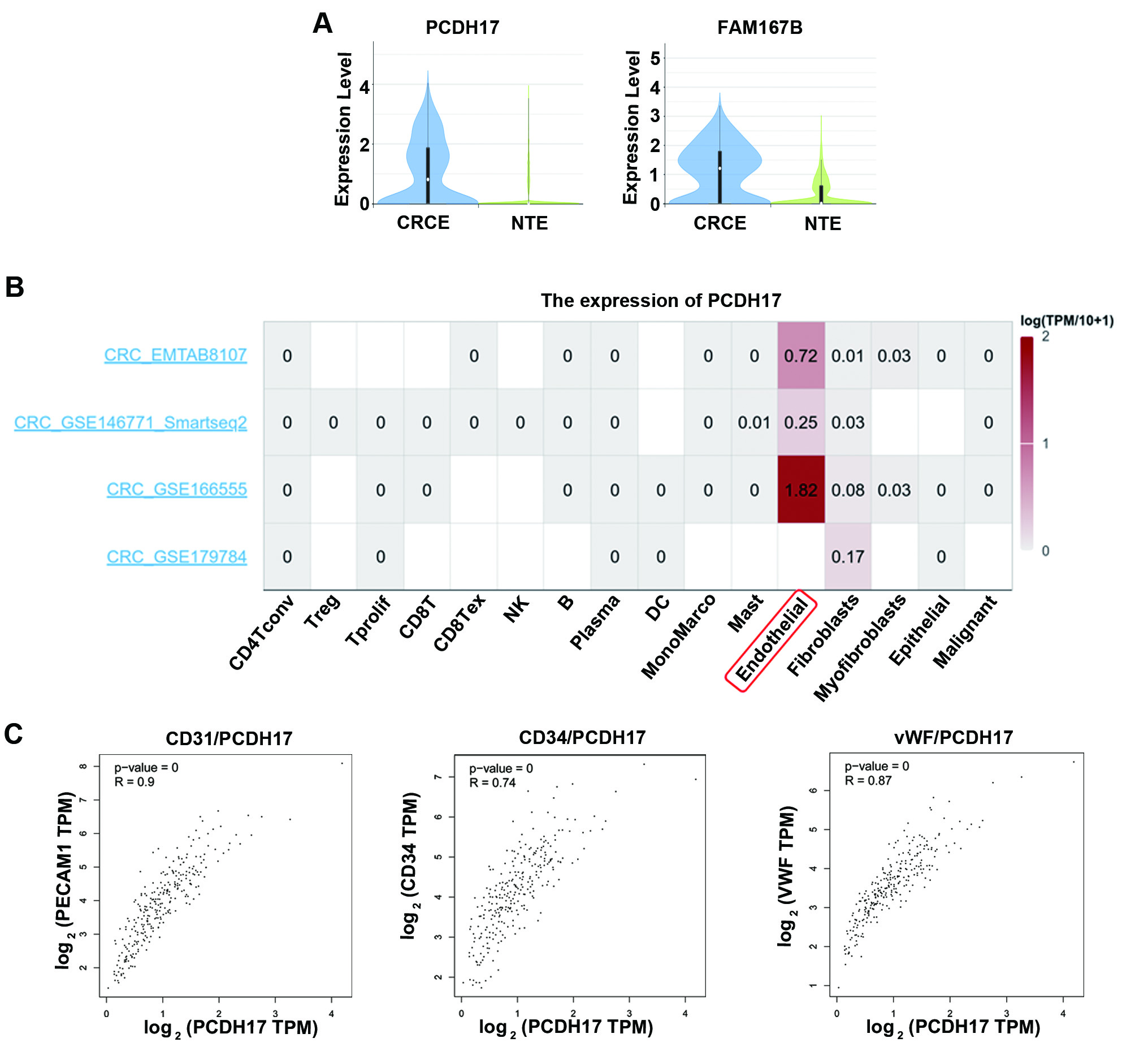

Supplement: Supplementary file 2 — Supplemental Figure S1 [file 41419_2025_7355_MOESM2_ESM.jpg]

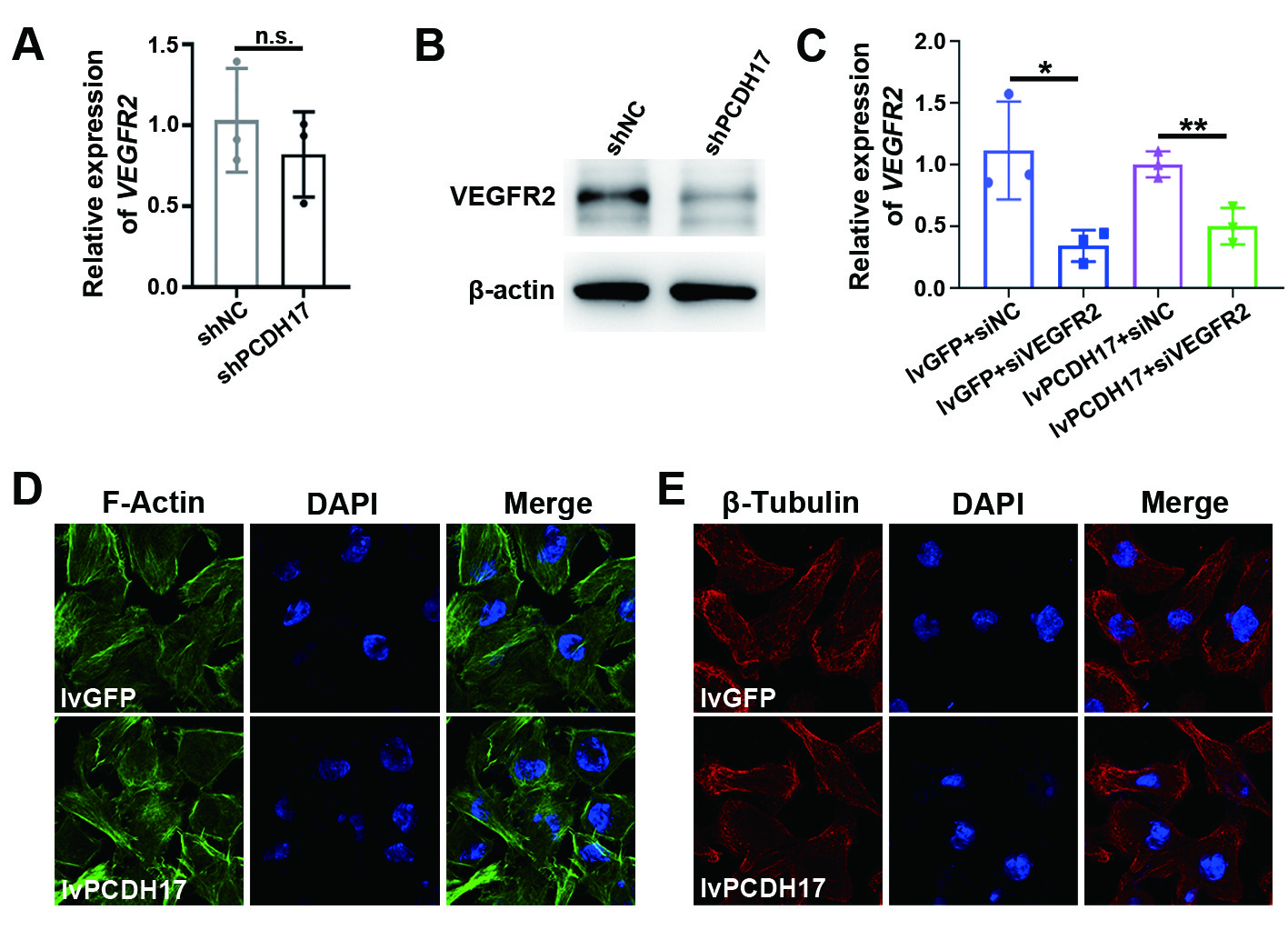

Supplement: Supplementary file 3 — Supplemental Figure S2 [file 41419_2025_7355_MOESM3_ESM.jpg]
