# Supplementary material for: PCDH17 induces colorectal cancer metastasis by destroying the vascular endothelial barrier
Source: Cell Death Dis. 2025 Jan 21;16(1):36. doi: 10.1038/s41419-025-07355-z (PMC11750977; doi:10.1038/s41419-025-07355-z)
Supplement: Supplementary file 7 — Supplemental Table S4 [file 41419_2025_7355_MOESM7_ESM.doc]

**Table S4: Targeting sequences of siRNAs**

| Gene | Sequence |
| --- | --- |
| VEGFR2 siRNA |  |
| Forward | GAG CAU GGA AGA GGA UUC UTT |
| Reverse | AGA AUC CUC UUC CAU GCU CTT |
| Control siRNA |  |
| Forward | UUCUCCGAACGUGUCACGUTT |
| Reverse | ACGUGACACGUUCGGAGAATT |

Note: All sequences are in the 5' to 3' orientation.
