# Supplementary material for: PCDH17 induces colorectal cancer metastasis by destroying the vascular endothelial barrier
Source: Cell Death Dis. 2025 Jan 21;16(1):36. doi: 10.1038/s41419-025-07355-z (PMC11750977; doi:10.1038/s41419-025-07355-z)
Supplement: Supplementary file 8 — Supplemental Table S5 [file 41419_2025_7355_MOESM8_ESM.doc]

**Table S5: qRT-PCR primer sequences**

| Gene | Sequence |
| --- | --- |
| PCDH17 |  |
| Forward | ATGAATGCCGAGTGCTTGGT |
| Reverse | GTGCTCTCGCTTGTCTTTTCC |
| PV1 |  |
| Forward | AGAACTCAGACCTCCAACGC |
| Reverse | TCTCCACCTTCTGTTTCGCC |
| VEGFR2 |  |
| Forward | GGTTGTGTATGTCCCACCCC |
| Reverse | GAGTGGTGCCGTACTGGTAG |
| GAPDH |  |
| Forward | GGAGCGAGATCCCTCCAAAAT |
| Reverse | GGCTGTTGTCATACTTCGCATGG |

Note: All sequences are in the 5' to 3' orientation.
