# Supplementary material for: PCDH17 induces colorectal cancer metastasis by destroying the vascular endothelial barrier
Source: Cell Death Dis. 2025 Jan 21;16(1):36. doi: 10.1038/s41419-025-07355-z (PMC11750977; doi:10.1038/s41419-025-07355-z)
Supplement: Supplementary file 9 — Supplemental Table S6 [file 41419_2025_7355_MOESM9_ESM.doc]

**Table S6. Identify PCR primer sequences for KO Mice**

| Gene | Sequence |
| --- | --- |
| Primer F1 |  |
| Forward | GCCCTCACAAAGGAACAATAACAGG |
| Reverse | ATTTGCTCCGGGTCTGATGATC |
| Primer F2 |  |
| Forward | CGAGATGGTTCAGTATCTGTAATGG |
| Reverse | AGCCAGAAGTCAGATGCTCAAGG |

Note: All sequences are in the 5' to 3' orientation.
