# Supplementary material for: PCDH17 induces colorectal cancer metastasis by destroying the vascular endothelial barrier
Source: Cell Death Dis. 2025 Jan 21;16(1):36. doi: 10.1038/s41419-025-07355-z (PMC11750977; doi:10.1038/s41419-025-07355-z)

Figure 3C

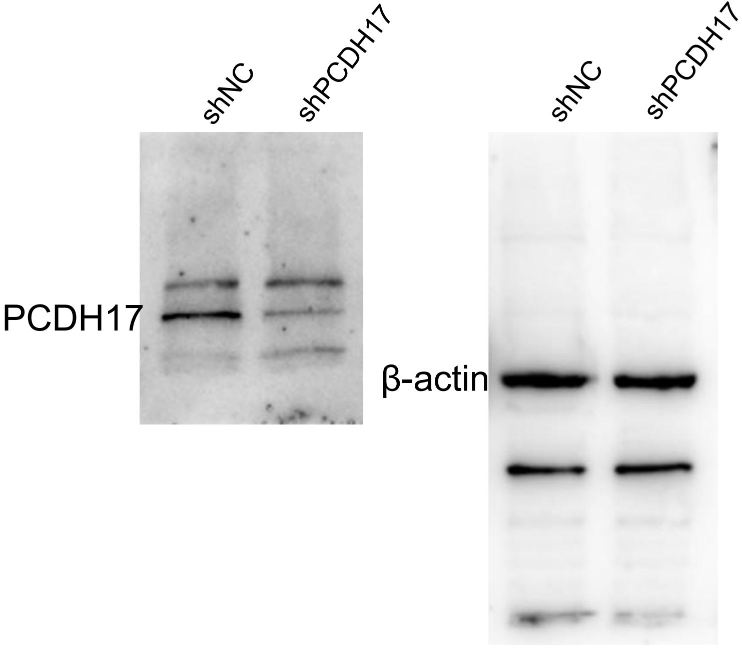

Figure 3D

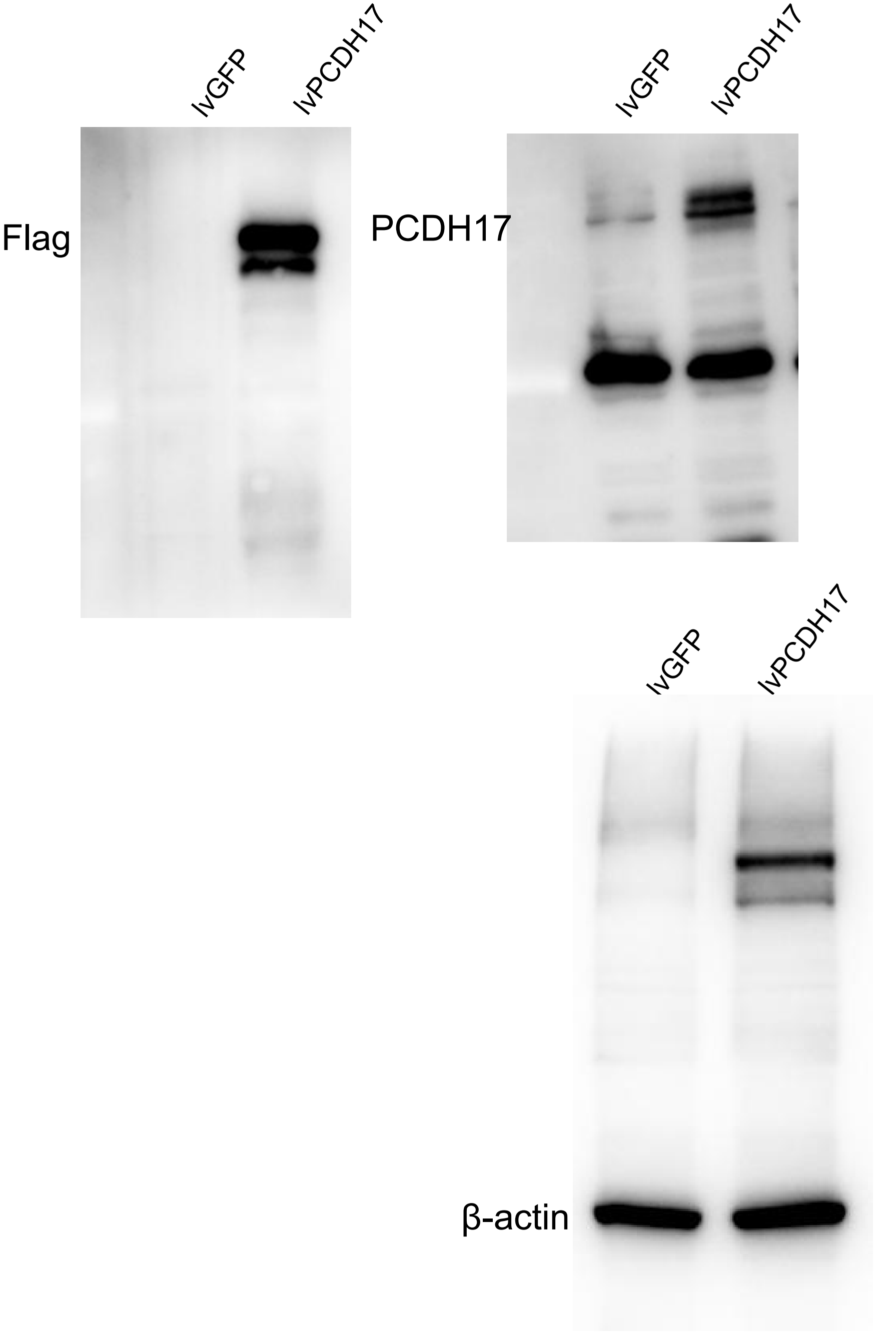

Figure 5B

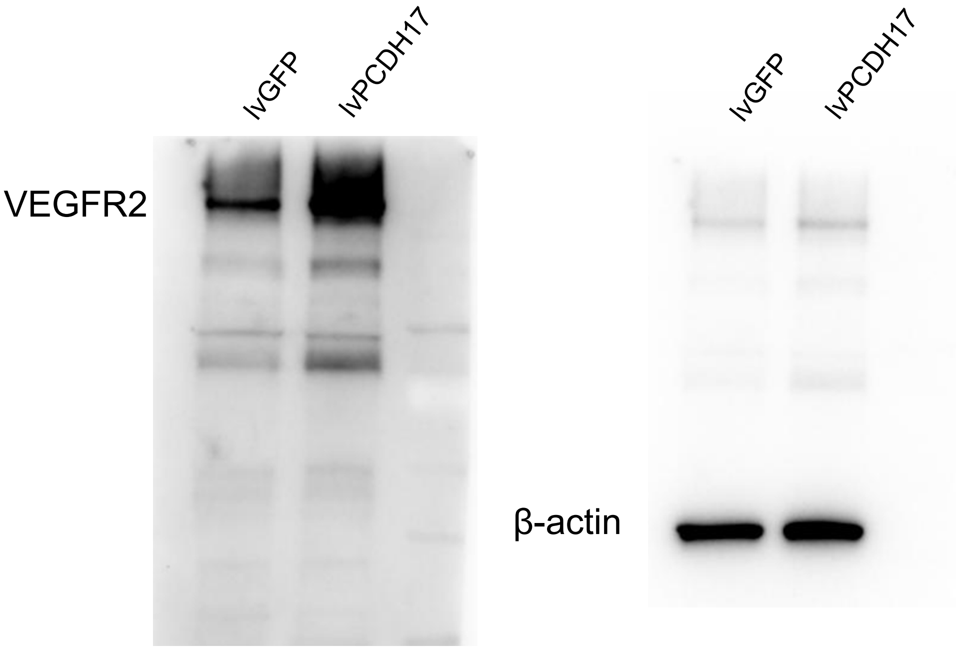

Figure 5C

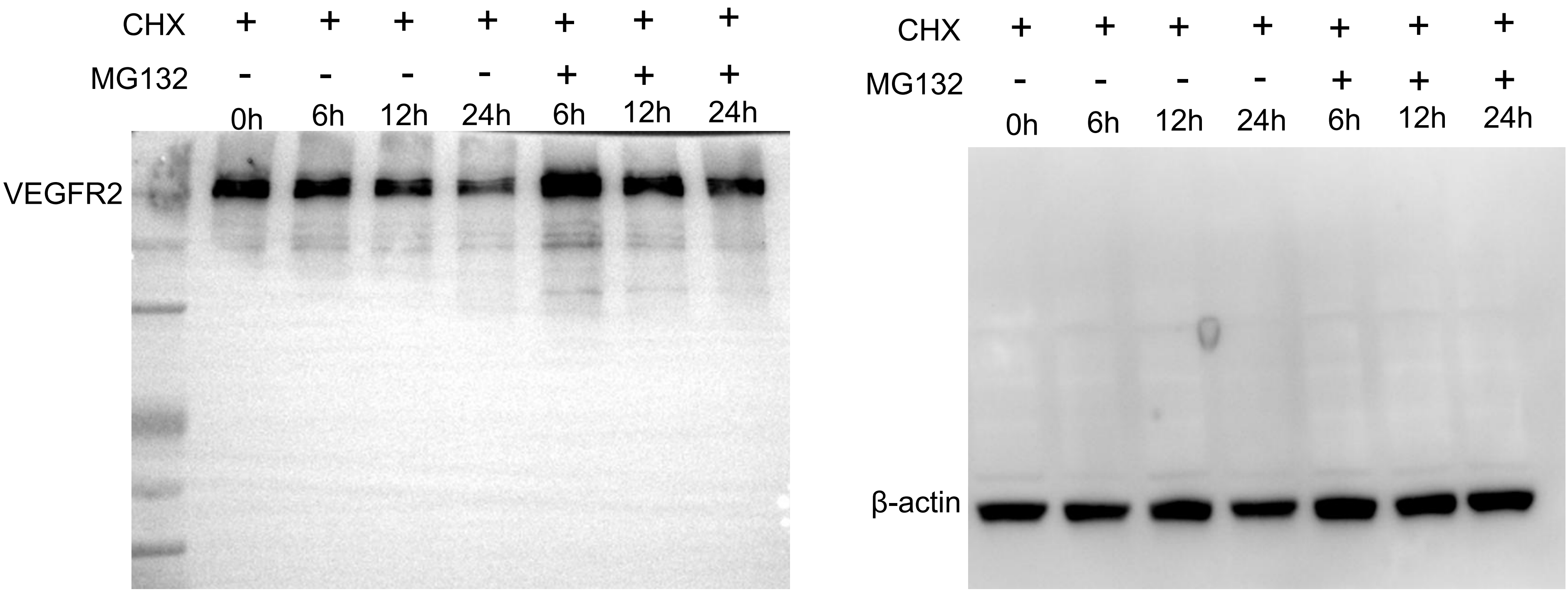
CHX + + + + + + +
MG132 - - - - + + +
0h 6h 12h 24h 6h 12h 24h
VEGFR2
CHX + + + + + + +
MG132 - - - - + + +
0h 6h 12h 24h 6h 12h 24h
β-actin

Figure 5D

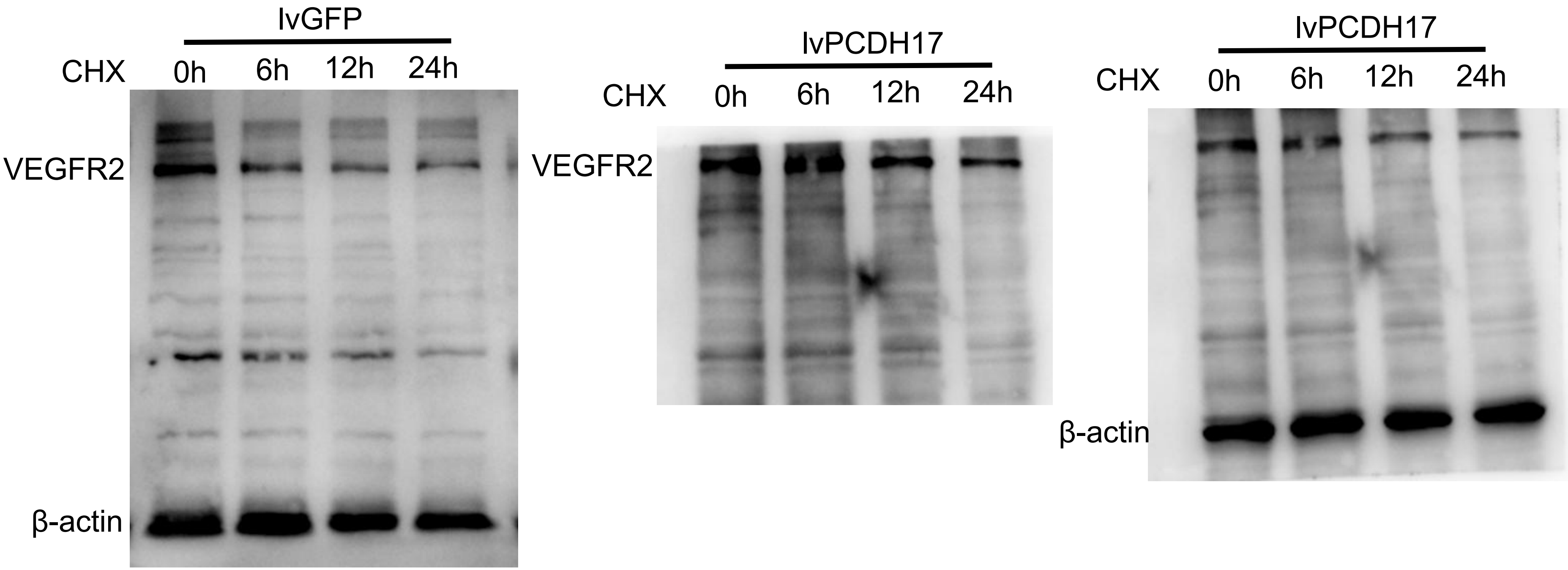
lvGFP
CHX 0h 6h 12h 24h
VEGFR2
β-actin
lvPCDH17
CHX 0h 6h 12h 24h
VEGFR2
lvPCDH17
CHX 0h 6h 12h 24h
β-actin

Figure 5G

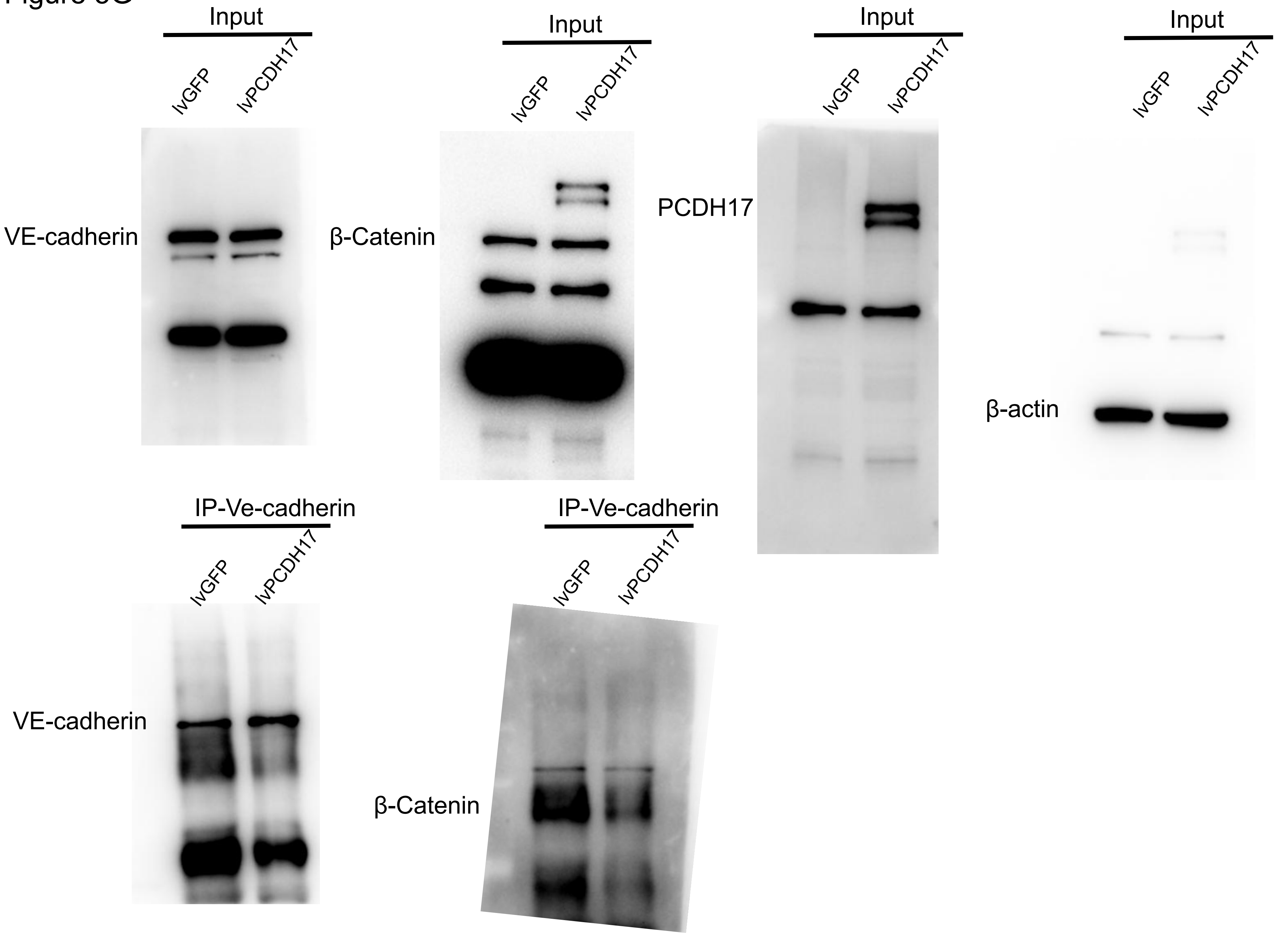

Input
lvGFP
lvPCDH17
VE-cadherin
Input
lvGFP
lvPCDH17
β-Catenin
Input
lvGFP
lvPCDH17
PCDH17
Input
lvGFP
lvPCDH17
β-actin
IP-Ve-cadherin
lvGFP
lvPCDH17
VE-cadherin
IP-Ve-cadherin
lvGFP
lvPCDH17
β-Catenin

Figure 6C

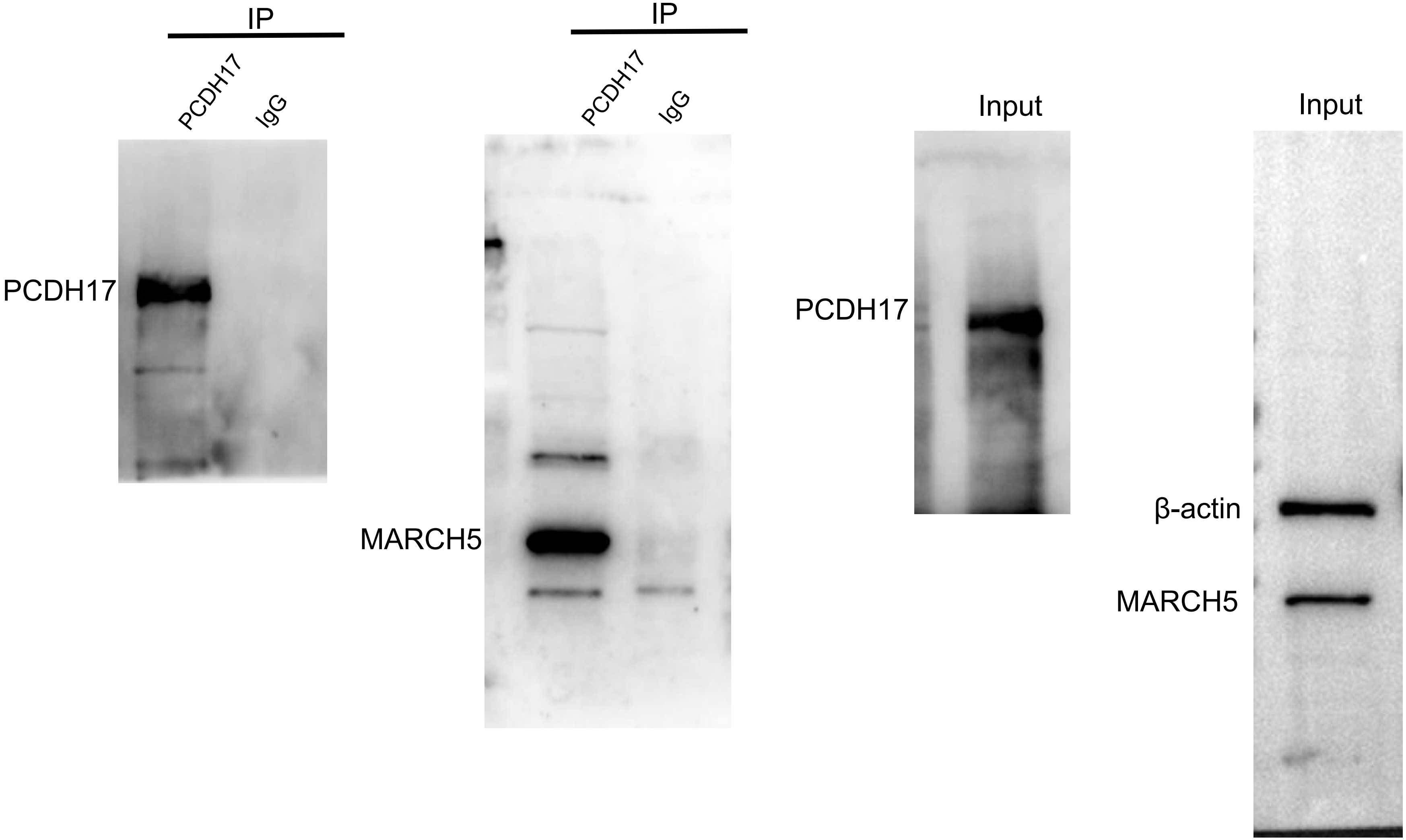
IP
PCDH17
IgG
PCDH17
IP
PCDH17
IgG
MARCH5
Input
PCDH17
Input
β-actin
MARCH5

Figure 6D

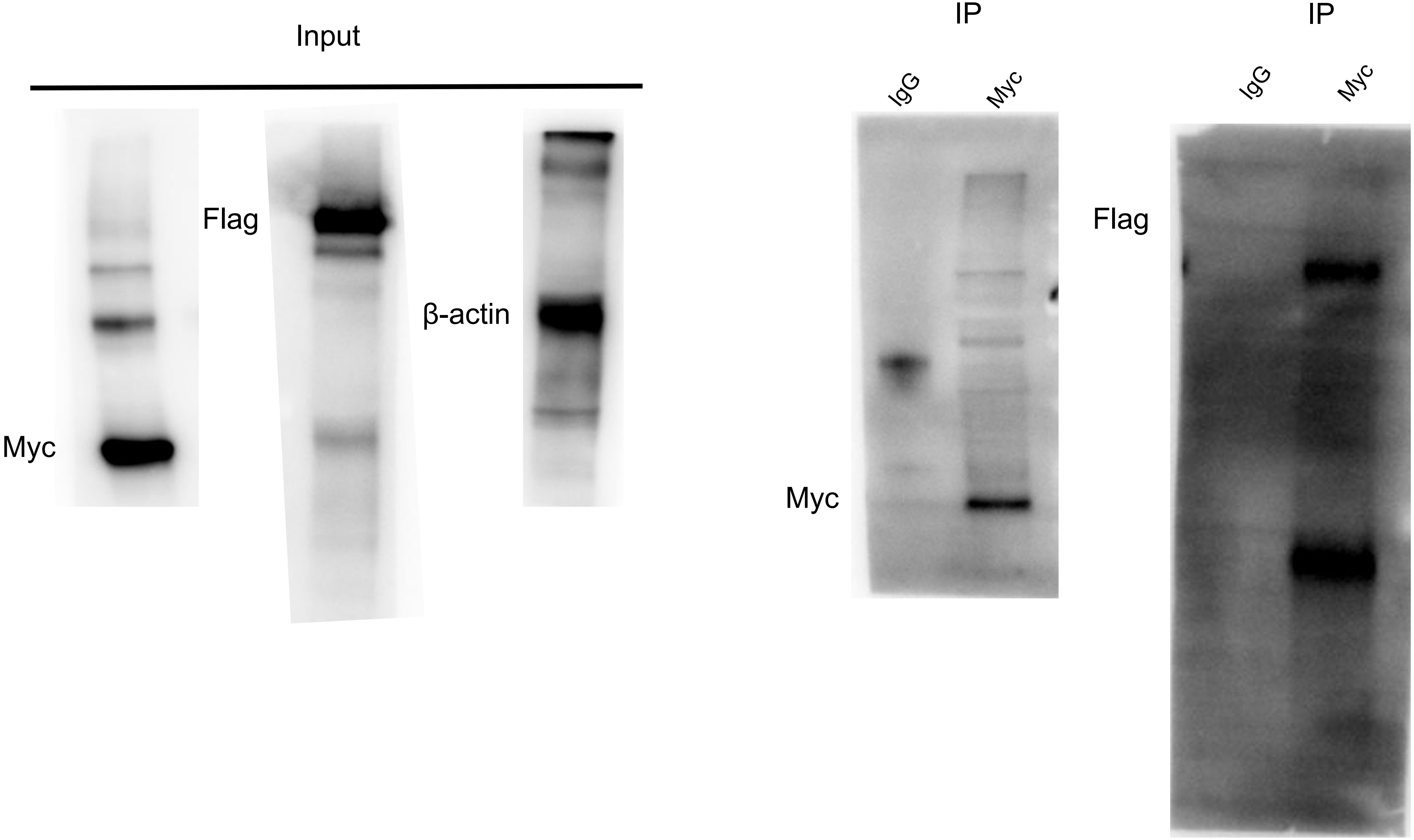

Figure 6E

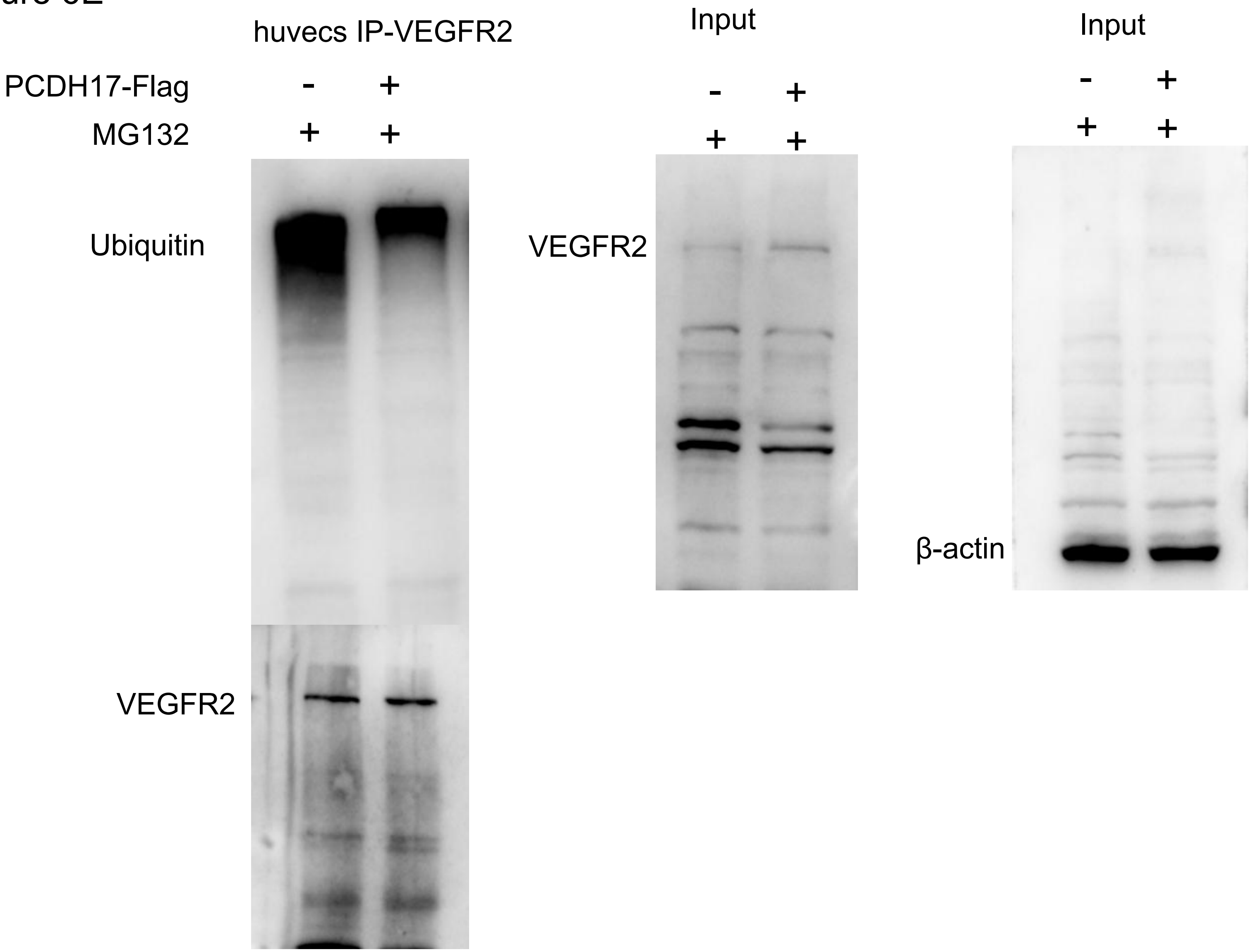

huvecs IP-VEGFR2
PCDH17-Flag
MG132
-
+
+
+
Ubiquitin
VEGFR2
Input
-
+
+
+
VEGFR2
Input
-
+
+
+
β-actin

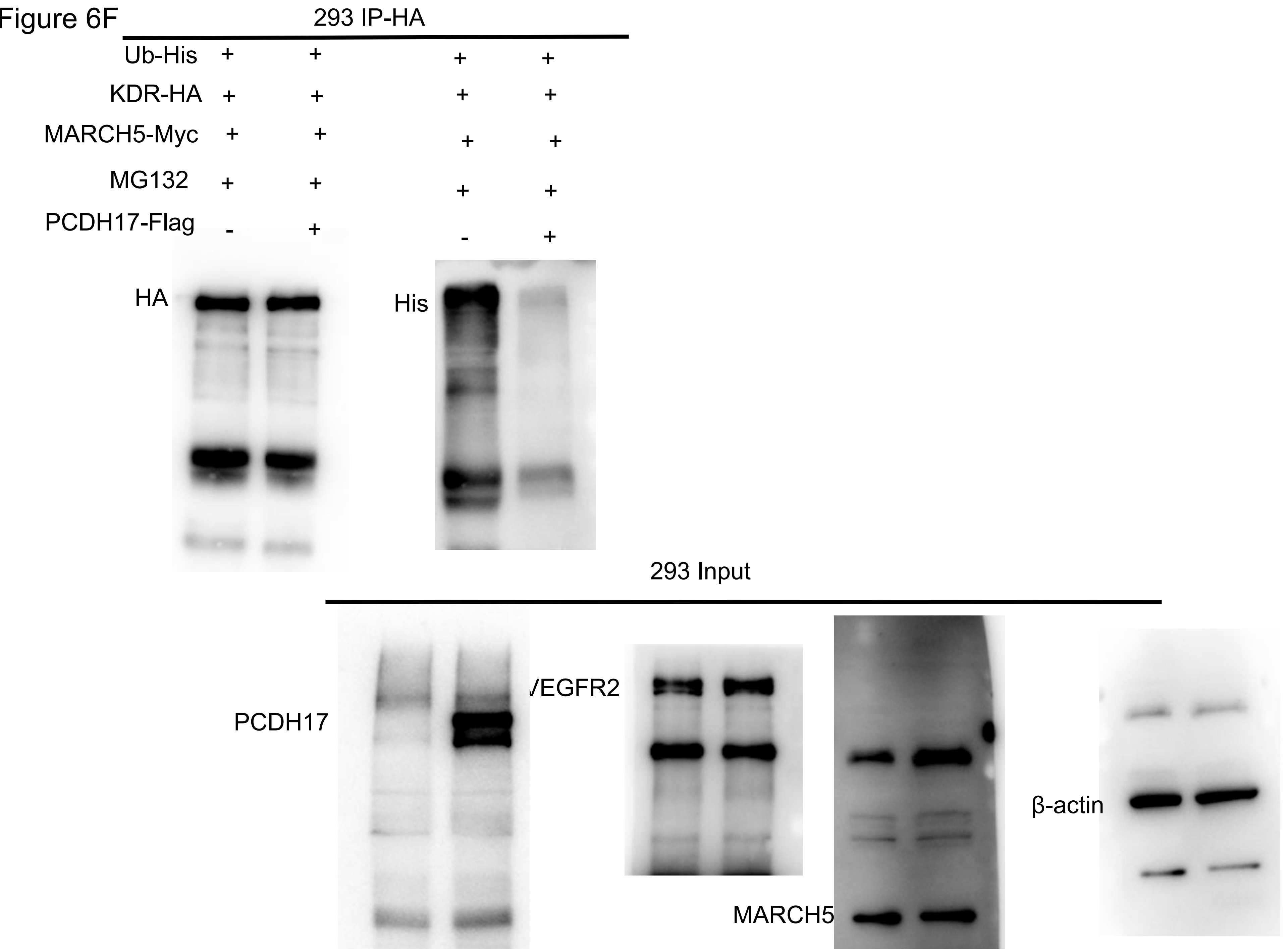
Figure 6F
293 IP-HA
Ub-His + + + +
KDR-HA + + + +
MARCH5-Myc + + + +
MG132 + + + +
PCDH17-Flag - + - +
HA
His
293 Input
PCDH17
VEGFR2
MARCH5
β-actin

Figure 6G

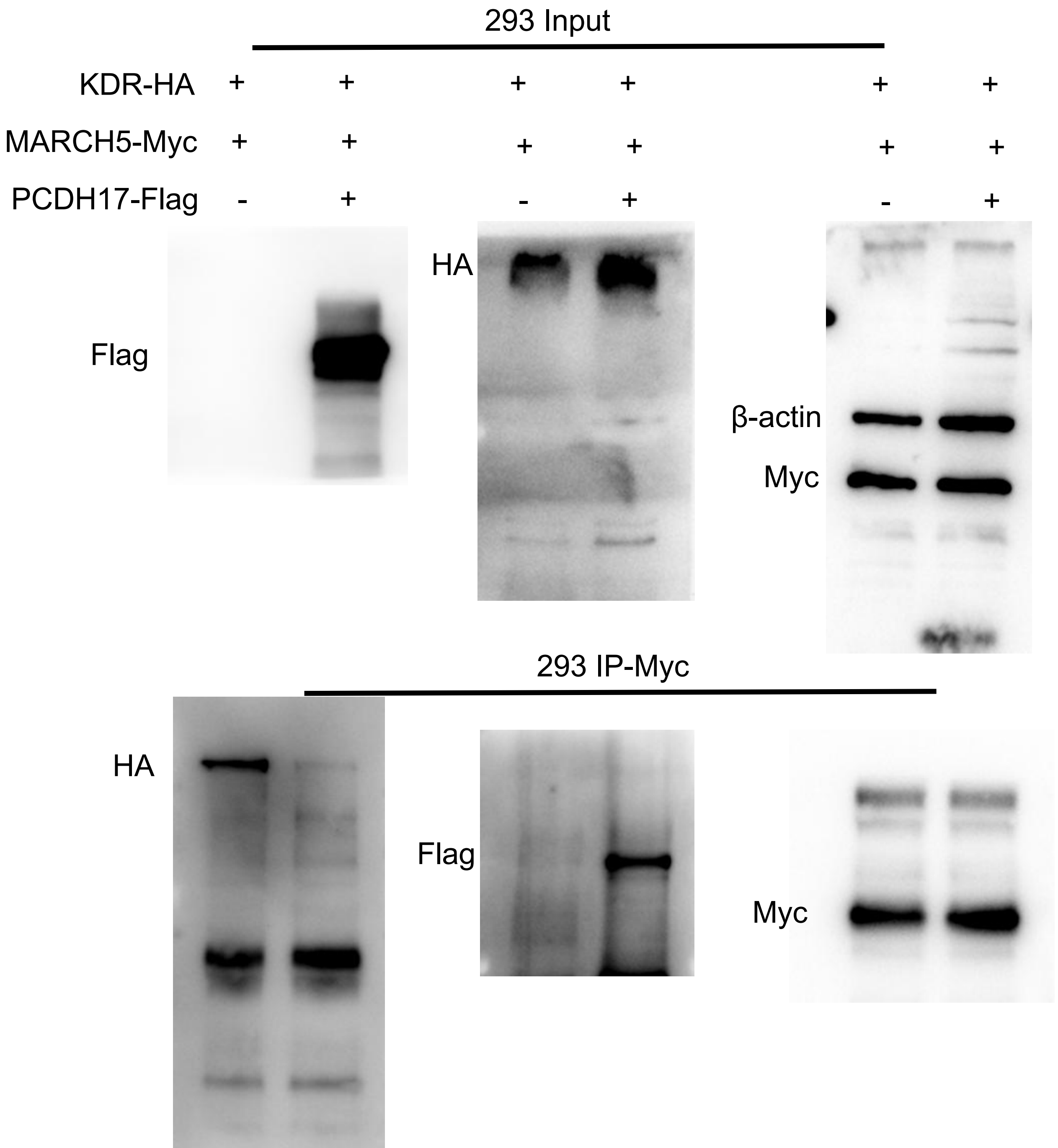
293 Input
KDR-HA + + + + + +
MARCH5-Myc + + + + + +
PCDH17-Flag - + - + - +
Flag
HA
β-actin
Myc
293 IP-Myc
HA
Flag
Myc

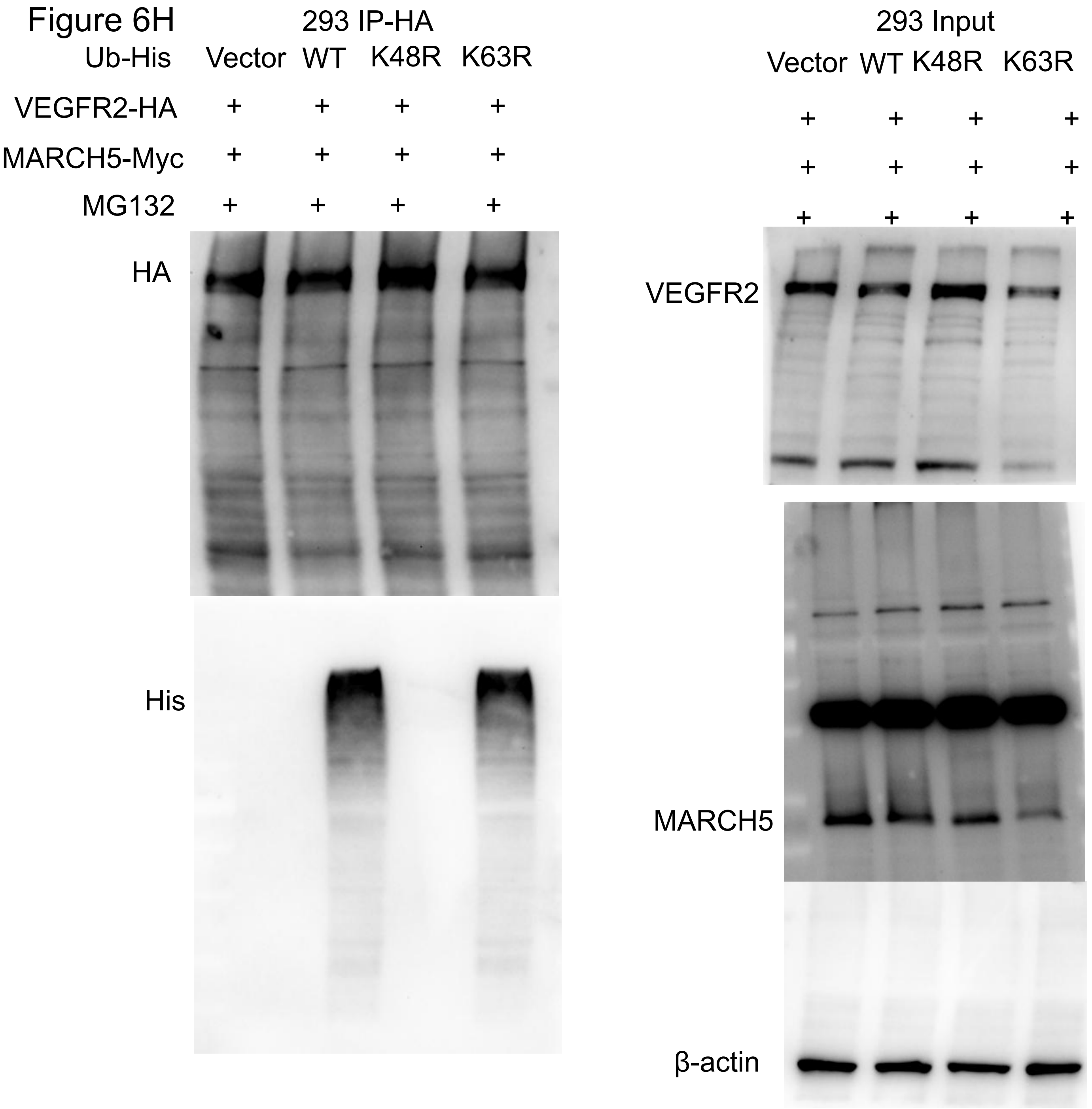

Figure 6H
293 IP-HA
Ub-His
Vector
WT
K48R
K63R
VEGFR2-HA
MARCH5-Myc
MG132
+
HA
His
293 Input
Vector
WT
K48R
K63R
VEGFR2
MARCH5
β-actin

Figure S2B

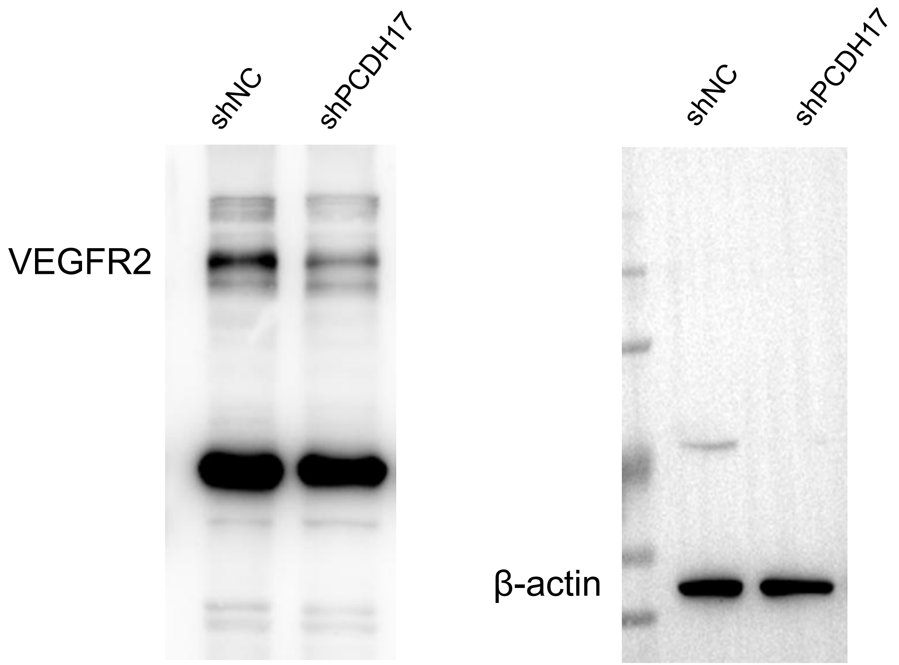

Supplement: Supplementary file 10 — Original data [file 41419_2025_7355_MOESM10_ESM.pdf]
